# Multi-temperature experiments to ease analysis of heterogeneous binder solutions by surface plasmon resonance biosensing

**DOI:** 10.1038/s41598-022-18450-y

**Published:** 2022-08-24

**Authors:** Jimmy Gaudreault, Yves Durocher, Olivier Henry, Gregory De Crescenzo

**Affiliations:** 1 Department of Chemical Engineering, Polytechnique Montréal, Centre-Ville Station, P.O. Box 6079, Montreal, QC H3C 3A7 Canada; 2 Life Sciences, NRC Human Health Therapeutics Portfolio, Building Montreal-Royalmount, National Research Council Canada, Montreal, QC H4P 2R2 Canada

**Keywords:** Biophysical chemistry, Assay systems

## Abstract

Surface Plasmon Resonance (SPR) biosensing is a well-established tool for the investigation of binding kinetics between a soluble species and an immobilized (bio)molecule. While robust and accurate data analysis techniques are readily available for single species, methods to exploit data collected with a solution containing multiple interactants are scarce. In a previous study, our group proposed two data analysis algorithms for (1) the precise and reliable identification of the kinetic parameters of N interactants present at different ratios in N mixtures and (2) the estimation of the composition of a given mixture, assuming that the kinetic parameters and the total concentration of all interactants are known. Here, we extend the first algorithm by reducing the number of necessary mixtures. This is achieved by conducting experiments at different temperatures. Through the Van’t Hoff and Eyring equations, identifying the kinetic and thermodynamic parameters of N binders becomes possible with M mixtures with M comprised between 2 and N and at least N/M temperatures. The second algorithm is improved by adding the total analyte concentration as a supplementary variable to be identified in an optimization routine. We validated our analysis framework experimentally with a system consisting of mixtures of low molecular weight drugs, each competing to bind to an immobilized protein. We believe that the analysis of mixtures and composition estimation could pave the way for SPR biosensing to become a bioprocess monitoring tool, on top of expanding its already substantial role in drug discovery and development.

## Introduction

Surface Plasmon Resonance (SPR)-based biosensors were first commercialized in the 1990s by Pharmacia. Since then, their ability to measure binding between an immobilized species and a solution species in real time without the need of any label has generated great interest in the biopharmaceutical field. Through repeated advances in the liquid handling and control systems^1–5^, the development of robust protocols^6^ and reproducible assays, as well as elaborate data analysis methods^7–15^, SPR biosensors have become a prominent tool for the study of biomolecular interactions and drug development.

With the recent implementation of the quality by design (QbD) and process analytical technology (PAT) frameworks^16–18^, quality monitoring tools have never been more needed. In this context, it appears appropriate to explore the potential of SPR biosensing for monitoring. Some SPR-based monitoring applications have already been reported for a variety of biotechnological applications (biotherapeutics, vaccines, bacterial detection)^19^. Still, to further establish SPR as a monitoring tool rather than only an investigation tool, new assays and data analysis algorithms will prove necessary.

In that endeavor, our group is interested in extending the use of SPR biosensing to the determination of the composition of biomolecule mixtures. For this purpose, a ligand that is common to all biomolecules in a mixture (the analytes) must be immobilized on the sensor surface. Then, reconstructing the signal recorded when injecting a mixture of analytes on the sensor surface, as a sum of the contribution of each analyte, should allow composition estimation. However, this requires prior knowledge of the analyte-specific kinetics for each analyte-ligand interaction. In cases where the analytes are easily purified, classical single-analyte experiments may be performed to uncover the kinetic behavior of each interaction. However, in cases where the biomolecules of interest are difficult to separate from one another (i.e., when they are only available as part of a mixture), advanced parameter identification algorithms must be employed to extract analyte-specific kinetics from multi-analyte experiments.

An example of such systems of biomolecules that form complex mixtures that are difficult to purify is the various glycoforms of therapeutic monoclonal antibodies that are produced via mammalian cell culture. The glycosylation state of these antibodies affects binding to their receptors^20–26^, as can be assessed by SPR^27^. Hence, advanced modeling techniques are required to analyze antibody sensorgrams and elucidate the kinetic behavior of the individual glycoforms. An SPR assay capable of monitoring the glycosylation profile of proteins would be of great interest for quality control purposes.

Apart from differences in glycosylation state, other potential applications of the multi-analyte framework include cases where molecules of different sizes or different bioactivities (due to cleavage or partial denaturation, for example) are present in the same solution and interact with the same ligand.

In a previous study^15^, we demonstrated that it is possible to identify the kinetic parameters of $N$ analytes with the same number of independent mixtures ($M$) of the analytes (i.e. $M=N$). Furthermore, we showed that the composition of an unknown mixture could be estimated using these identified kinetic parameters. These methods were validated using experimental data obtained by immobilizing the enzyme carbonic anhydrase II (CAII) and injecting mixtures of four CAII binders. Two main limitations remained in this framework. First, the number of available mixtures might be limiting when $N$ is large. Second, the composition estimation algorithm required prior knowledge of the total concentration of all analytes combined, which is not always readily available.

To address these limitations, the present study suggests two new algorithms that will be applied on the same system as in^15^. The first algorithm aims to reduce the number of necessary mixtures by injecting the available mixtures at different temperatures. A priori, kinetic rates and affinities of different analytes will exhibit different temperature dependencies. In brief, the information that is lacking because $M<N$ is replaced by surveying at different temperatures. As the kinetics and affinities are evaluated at different temperatures, thermodynamic analyses via the Eyring and Van’t Hoff equations can be performed. These equations have previously been used to evaluate enthalpic and entropic changes via single-analyte SPR experiments with CAII binders^28^. The second algorithm presented here aims to estimate both the composition and the concentration of an unknown mixture of analytes, assuming prior knowledge of their kinetics. Essentially, the concentration is added as a supplementary parameter to be identified along with the analyte fractions. Only sensorgrams recorded at one temperature are necessary for our second algorithm. Therefore, we also included a guideline on how to choose the optimal injection temperature for composition estimation. These two algorithms were validated with the previously mentioned CAII system.

## Materials and methods

### Materials

Biacore T100 biosensor, research-grade CM5 sensor chips, HBS-EP buffer (HEPES Buffered Saline with 30 mM EDTA [ethylenediaminetetraacetic acid] and 0.5% (v/v) surfactant P20), 70% v/v glycerol in water and ethanolamine were purchased from Cytiva (formerly GE Healthcare, Marlborough, MA). N-ethyl-N’-(3-dimethylaminopropyl) carbodiimide (EDC), N-hydroxysuccinimide (NHS), carbonic anhydrase isozyme II (CAII) purified from bovine erythrocytes, glacial acetic acid, sodium acetate, 4-carboxybenzenesulfonamide (CBS), 1,3-benzene-disulfonamide (BDS), sulfanilamide, furosemide and dimethyl sulfoxide (DMSO) were purchased from Sigma-Aldrich Canada Ltd (Oakville, ON).

### Biosensor surface preparation

The CM5 sensor chips used in this study are composed of gold on which carboxymethylated dextran has been grafted by the chip supplier. The biosensor surface preparation was performed according to a previously published protocol^29^. In short, two sensor chip surfaces were activated at 25 °C with an injection of 1:1 (v/v) 0.4 M EDC and 0.1 M NHS during 7 min at 20 μL/min. A solution of 0.1 g/L of CAII was prepared in a 10 mM acetate buffer at pH 5.0. This solution was injected on only one of the surfaces (experimental surface) through 30 s pulses at 20 μL/min until a density of immobilized CAII of approximately 5000 Resonance Units (RU) was reached. Next, the remaining active sites were blocked by injecting ethanolamine (1 M at pH 8.5) at 20 μL/min during 4 min. This procedure led to an immobilized CAII density of approximately 4500 RU. For each CAII surface, a mock surface (for referencing purposes) was generated by activating/deactivating the surface with the same solutions (without any CAII injections). Before and after the preparation of the surfaces, the system was extensively primed with running buffer (HBS-EP containing 3% of DMSO) and a normalization procedure with a solution of 70% (v/v) glycerol in water was performed after ligand immobilization. For more information on SPR biosensors inner-workings, we refer the reader to relevant reviews^1–5^.

### SPR experiments

#### Analyte preparation

HBS-EP containing 3% of DMSO (HBS-EP + 3%DMSO) was used as the running buffer. All the analytes were dissolved in DMSO and aliquoted at the following concentrations: [CBS] = 596.42 mM, [BDS] = 118.51 mM, [sulfanilamide] = 563.30 mM, [furosemide] = 3075.30 mM. For each experiment, one aliquot of each analyte was dissolved in HBS-EP + 3%DMSO to reach the following concentrations: [CBS] = 5.96 mM, [BDS] = 1.19 mM, [sulfanilamide] = 5.63 mM, [furosemide] = 30.75 mM. These stock solutions were then used in the preparation of the different dilutions and mixtures analyzed in this study.

#### Single-analyte experiments

For classical single-analyte kinetic experiments, CBS, BDS, sulfanilamide and furosemide were injected alone at 7 different concentrations (two-fold dilutions) ranging from 350 nM to 22 400 nM for CBS and furosemide, 175 nM to 11 200 nM for BDS and 900 nM to 57 600 nM for sulfanilamide. All injections were performed in duplicates and both repetitions were used separately in the analysis. The flow rate was 85 μL/min and the data collection rate was 10 Hz. Analytes were injected at four different temperatures: 12, 16, 20 and 24 °C. For double-referencing purposes, 4 buffer injections per analyte per temperature were also performed. The injection time was set to 240 s for all analytes and the dissociation time of the resulting complexes ranged from 270 to 570 s and was adjusted for each analyte and temperature such that the SPR signal reaches zero towards the end of the dissociation phase. Hence, no regeneration step was needed in between analyte injections as the dissociation was complete in every case. This agrees with various previous studies in which this system was analyzed via SPR^10–12,15,29,30^. The SPR biosensor used in this study (Biacore T100) uses the geometry proposed by Kretschmann and Raether^1,3^. The detection is performed by varying the incident angle while keeping the wavelength constant.

#### Multiple-analyte experiments

The four analytes were combined to create four random mixtures A to D, as described in Table 1. The mixture compositions were chosen randomly while ensuring that all mixtures contain all the analytes, and that no analyte has a dominating presence in any of the mixtures. All mixtures were injected at four different temperatures: 12, 16, 20 and 24 °C. In every case, the association phase duration was set to 240 s and the dissociation time was adjusted such that null responses were obtained at the end of the sensorgrams (ranging from 270 to 570 s), circumventing the need for a regeneration step. The injection flow rate was set to 85 μL/min and the data collection rate was 10 Hz. The mixtures were injected in two-fold dilutions ranging from 350 nM to 22 400 nM.

**Table 1:** Molar composition of the analyte solutions used in this study.

Analyte solution	% CBS	% BDS	% Sulfanilamide	% Furosemide
CBS	100	0	0	0
BDS	0	100	0	0
Sulfanilamide	0	0	100	0
Furosemide	0	0	0	100
A	40	10	30	20
B	15	25	50	10
C	20	40	10	30
D	17	21	10	52

#### Furosemide standard injections

The CAII surface may wear off in time as more and more experiments are performed. This can cause a loss of activity of the immobilized ligand molecules, leading to responses with slightly lower amplitudes. To account for this loss, the sensorgrams were standardized. Before the dilution series of a given mixture was injected at a given temperature, a standard furosemide injection was performed. This was repeated for each mixture and each injection temperature. This allowed to monitor the condition of the CAII surface. A saturating concentration of furosemide was used for the standard (51 480 nM). Injections lasted 60 s, which was long enough to reach equilibrium at every temperature. The value of the plateau (standard value) was extracted. The standardization process was performed for a given sensorgram by comparing the standard value of the previous standard injection with that of the very first standard injection:

1 $$Respons{e}_{standardized}=Response\cdot \frac{Standar{d}_{First}}{Standar{d}_{Previous}}$$

Standard injections were also double-referenced according to the procedure described in^6^.

#### Data Analysis

Data analysis algorithms for single- and multi-analyte experiments assumed that each analyte bound to CAII according to a 1:1 Langmuir model with a constant theoretical maximal response and temperature dependent kinetic rate constants. In the case of classical single-analyte experiments, identification of the interaction kinetics was performed simultaneously for all temperatures. Thus, the maximal response of a given analyte was assumed to be constant for all temperatures. Adding this constraint did not significantly alter the identified parameters compared to fitting the model independently for each temperature: deviations of the order of 5% were observed compared to independent fits. This procedure was performed with a custom-designed script in the Matlab R2018b software platform (The Mathworks, Natick, USA). Parameters obtained with our procedure also matched those obtained with the Biacore T100 Evaluation Software^31^.

We developed two algorithms. The first one aims to extract the temperature dependent kinetic parameters from a set of sensorgrams corresponding to various analyte mixtures (Table 1) injected at different temperatures. This also allows the identification of thermodynamic parameters. The second algorithm estimates the composition of a given mixture and its concentration based on the prior knowledge of the kinetic (and thermodynamic) constants of each analyte. Both algorithms are presented in the next section. Fitting the multi-analyte data and estimating mixture composition was also performed using in-house Matlab scripts which are available upon request. Once the recorded sensorgrams have been retrieved from the Biacore Evaluation Software and inputted in the Matlab environment, the rest of the method is automatic, be it for parameter identification or unknown mixture analysis. As sensorgrams corresponding to the different dilutions of the different mixtures injected at different temperatures needed to be analyzed simultaneously within the fitting procedure, this type of analysis will be further described in this research as the 'global-global-global' approach.

## Theory

Our first aim is to extract kinetic parameters ${k}_{a,i} \left[=\right] {s}^{-1}.{M}^{-1}$ and ${k}_{d,i} \left[=\right] {s}^{-1}$ and maximum response amplitude ${R}_{max,i}= \left[=\right] RU$ ($i=\mathrm{1,2},\dots N$) of $N$ analytes from $M<N$ mixtures with known proportions of each analyte ${F}_{i}$. To achieve this, the mixtures will be injected on a SPR surface harboring a common ligand that binds all the analytes. Injections will be performed at different temperatures. Hence, association and dissociation rates (${k}_{a,i}$ and ${k}_{d,i}$) will be obtained for multiple temperatures. This will allow the identification of thermodynamic parameters that describe the temperature dependence of ${k}_{a,i}$ and ${k}_{d,i}$ which follows the Eyring equation. Knowing ${k}_{a,i}$ and ${k}_{d,i}$, the affinity of the analyte-ligand interaction (${K}_{A,i}$) is calculated: ${K}_{A,i}=\frac{{k}_{a,i}}{{k}_{d,i}} \left[=\right] {M}^{-1}$. Temperature dependence of ${K}_{A,i}$ is described with the Van’t Hoff equation. Both the Eyring and Van’t Hoff equations were previously used with the present analyte-ligand system in a single-analyte experiment context^28^.

Our second aim is to use the identified parameters in order to estimate the composition of an unknown mixture of the $N$ analytes, that is the proportion of each analyte in the mixture. A procedure has already been proposed by our group for the case where the total concentration of all analytes combined is known^15^. Here, we suggest an extension of this method for the case where the total concentration is also unknown. Ultimately, both the composition and the concentration will be determined.

### Multi-Analyte SPR Model

We assume a Langmuir 1:1 interaction between each analyte (${A}_{i}$) and the ligand ($L$):

2 $${A}_{i}+L\begin{array}{c}\stackrel{{k}_{a,i}}{\to }\\ \underset{{k}_{d,i}}{\leftarrow }\end{array}{A}_{i}L, \forall i=1,\dots ,N$$

With ${k}_{a,i}$ and ${k}_{d,i}$ corresponding to the association and dissociation rate constants of the ${i}^{th}$ analyte, respectively. For $N$ analytes, the system is described by the following ordinary differential equation (ODE) system:

3 $$\begin{array}{l}\frac{\mathrm{d}{R}_{i}}{\mathrm{d}t}={k}_{a,i}{F}_{i}{C}_{TOT}{R}_{max,i}\left(1-\sum_{j=1}^{N}\frac{{R}_{j}}{{R}_{max,j}}\right)-{k}_{d,i}{R}_{i}, {R}_{i}\left(0\right)=0, \forall i=1,\dots ,N\\ {R}_{TOT}=\sum_{i=1}^{N}{R}_{i}\end{array}$$

${R}_{max,i}$ is the theoretical SPR response that would be obtained if an infinite concentration of analyte $i$ were injected. ${F}_{i}$ is the fraction of analyte $i$ in the injected mixture and ${C}_{TOT}$ is the total concentration of all analytes. We can then compute the predicted SPR response (${R}_{TOT}$) and the response caused by each analyte (${R}_{i}$) by solving the system of ODE in (3).

#### Eyring Equation

The Eyring equation describes the evolution of a kinetic parameter ($k$ standing for either ${k}_{a}$ or ${k}_{d}$) with respect to temperature ($T$):

4 $$\begin{aligned}k(T)&=\left(\frac{{k}_{B}T}{h}\right)\mathrm{exp}\left(-\frac{\Delta {G}^{*}}{{R}_{IG}T}\right)\\k(T)&=\left(\frac{{k}_{B}T}{h}\right)\mathrm{exp}\left(-\frac{\Delta {H}^{*}}{{R}_{IG}T}+\frac{\Delta {S}^{*}}{{R}_{IG}}\right)\\k(T)&=\left(\frac{{k}_{B}T}{h}\right)\mathrm{exp}\left(\frac{\Delta {S}^{*}}{{R}_{\mathit{IG}}}\right)\mathrm{exp}\left(-\frac{\Delta {H}^{*}}{{R}_{IG}T}\right)\end{aligned}$$

$\Delta {G}^{*}=\Delta {H}^{*}-T\Delta {S}^{*} \left[=\right] cal.mo{l}^{-1}$ is the Gibbs energy of activation, and $\Delta {H}^{*} \left[=\right] cal.mo{l}^{-1}$ and $\Delta {S}^{*} \left[=\right] cal.mo{l}^{-1}.{K}^{-1}$ are the activation enthalpy and entropy for the pseudo-reaction describing the analyte-ligand complex formation (${k}_{a}$) or dissociation (${k}_{d}$). $\Delta {H}^{*}$ and $\Delta {S}^{*}$ are assumed to be temperature independent. ${R}_{IG}$ is the ideal gas constant, and ${k}_{B}$ and $h$ are the Boltzmann and Planck constants, respectively: ${k}_{B}=1.381*{10}^{-23}J.{K}^{-1}$ and $h= 6.626*{10}^{-34} J.s$.

The Eyring equation can be rewritten in simpler terms for both kinetic parameters by introducing constants ${C}_{1}$ through ${C}_{4}$:

5 $$\begin{aligned}{k}_{a,i}(T)&=T {C}_{1,i}\;\mathrm{exp}\left(\frac{{C}_{2,i}}{T}\right)\\{k}_{d,i}(T)&=T {C}_{3,i}\;\mathrm{exp}\left(\frac{{C}_{4,i}}{T}\right)\\{C}_{1,i}&=\frac{{k}_{B}}{h}\;\mathrm{exp}\left(\frac{\Delta {S}_{{k}_{a}}^{*}}{{R}_{IG}}\right).c= \left[=\right] {K}^{-1}.{s}^{-1}.{M}^{-1}\\{C}_{2,i}&=-\frac{{\Delta H}_{{k}_{a}}^{*}}{{R}_{IG}} \left[=\right] K\\{C}_{3,i}&=\frac{{k}_{B}}{h}\;\mathrm{exp}\left(\frac{\Delta {S}_{{k}_{d}}^{*}}{{R}_{IG}}\right)= \left[=\right] {K}^{-1}.{s}^{-1}\\{C}_{4,i}&=-\frac{{\Delta H}_{{k}_{d}}^{*}}{{R}_{IG}} \left[=\right] K\end{aligned}$$

$c=1 {M}^{-1}$ is an added factor to account for the fact that the association pseudo-reaction is not unimolecular. A priori, activation enthalpies and entropies are analyte-specific, which explains the presence of $i=\mathrm{1,2},\dots N$ indices in (5).

#### Contribution to the SPR response at equilibrium

The SPR response caused by analyte $i$ at equilibrium, noted ${R}_{eq,i} \left[=\right] RU$, is the amplitude of the response related to the presence of analyte $i$ near the SPR surface when the response has reached a plateau during the association rate. The response of every analyte at equilibrium is given by:

6 $${R}_{eq,i}=\frac{{F}_{i}{K}_{A,i}{R}_{max,i}{C}_{TOT}}{1+\sum_{i=1}^{N}{F}_{i}{K}_{A,i}{C}_{TOT}}=\frac{{F}_{i}{K}_{A,i}{R}_{max,i}{C}_{TOT}}{1+{K}_{A,obs}{C}_{TOT}} \forall i=\mathrm{1,2},\dots ,N$$

The total equilibrium response of a mixture of analytes (${R}_{eq,TOT} \left[=\right] RU$) is analogous to the response of a single analyte with affinity ${K}_{A,obs}$ and maximum response ${R}_{max,obs}$:

7 $${R}_{eq,TOT}=\sum_{i=1}^{N}{R}_{eq,i}=\frac{\sum_{i=1}^{N}{F}_{i}{K}_{A,i}{R}_{max,i}{C}_{TOT}}{1+{K}_{A,obs}{C}_{TOT}}=\frac{{K}_{A,obs}{R}_{max,obs}{C}_{TOT}}{1+{K}_{A,obs}{C}_{TOT}}$$

For a given mixture and temperature, ${K}_{A,obs}$ and ${R}_{max,obs}$ are identified using the observed plateau values for several injections at varying concentrations. These observed parameters are then linked to the analyte-specific parameters in the following way:

8 $${K}_{A,obs}=\sum_{i=1}^{N}{F}_{i}{K}_{A,i} \left[=\right] {M}^{-1}$$

9 $${R}_{max,obs}=\frac{\sum_{i=1}^{N}{F}_{i}{K}_{A,i}{R}_{max,i}}{{K}_{A,obs}} \left[=\right] RU$$

We denote ${Z}_{i}$ the contribution to the SPR response at equilibrium of analyte $i$ (dimensionless). It represents the fraction of the total response that is caused by a given analyte:

10 $$\begin{aligned}&{Z}_{i}=\frac{{R}_{eq,i}}{{R}_{eq,TOT}}=\frac{{F}_{i}{K}_{A,i}{R}_{max,i}}{{K}_{A,obs}{R}_{max,obs}}\\ &\sum_{i=1}^{N}{Z}_{i}=1 \end{aligned}$$

#### Analysis of multiple mixtures

If more than one mixture of the same analytes is available, the contribution of analyte $i$ in mixture ${m}_{1}$ is linked to that of the same analyte in another mixture ${m}_{2}$:

11 $${Z}_{i,{m}_{2}}={Z}_{i,{m}_{1}}\frac{{F}_{i,{m}_{2}}}{{F}_{i,{m}_{1}}}\frac{{\left({R}_{max,obs}{K}_{A,obs}\right)}_{{m}_{1}}}{{\left({R}_{max,obs}{K}_{A,obs}\right)}_{{m}_{2}}}$$

#### Analysis of multiple injection temperatures

If the mixtures were injected at more than one temperature, the dissociation kinetic at temperature ${T}_{1}$ is linked to that of another temperature ${T}_{2}$, via the Eyring Eq. (5):

12 $${k}_{d,i,{T}_{2}}={k}_{d,i,{T}_{1}}\frac{{T}_{2}}{{T}_{1}}\mathrm{exp}\left({C}_{4,i}\left(\frac{1}{{T}_{2}}-\frac{1}{{T}_{1}}\right)\right)$$

#### Response during the dissociation phase

For mixture $m$ injected at temperature $T$, if one assumes equilibrium was reached during the association phase, the SPR response during the dissociation phase (${R}_{tot}\left(t\right)$) is mathematically described by the following sum of exponentials:

13 $${R}_{tot,m,T}\left(t\right)=\sum_{i=1}^{N}{R}_{eq,i,m}\mathrm{exp}\left(-{k}_{d,i,T} t\right)$$

To introduce the equilibrium contributions (${Z}_{i,m}$), we divide the SPR response at each time step $t$ by the equilibrium plateau value ${R}_{tot}\left({t}_{diss}\right)$ with ${t}_{diss}$ indicating the beginning of the dissociation phase:

14 $${R}_{norm,m,T}\left(t\right)=\frac{{R}_{tot}\left(t\right)}{{R}_{tot}\left({t}_{diss}\right)}=\sum_{i=1}^{N}{Z}_{i,m}\mathrm{exp}\left(-{k}_{d,i,T} t\right)$$

This eliminates the effect of concentration: the normalized sensorgrams (${R}_{norm}$, dimensionless) corresponding to different analyte concentrations will be overlaid. A minimum of two concentrations is still necessary so that ${K}_{A,obs}$ and ${R}_{max,obs}$ may be identified using the equilibrium values.

### Algorithm

**Figure 1:**
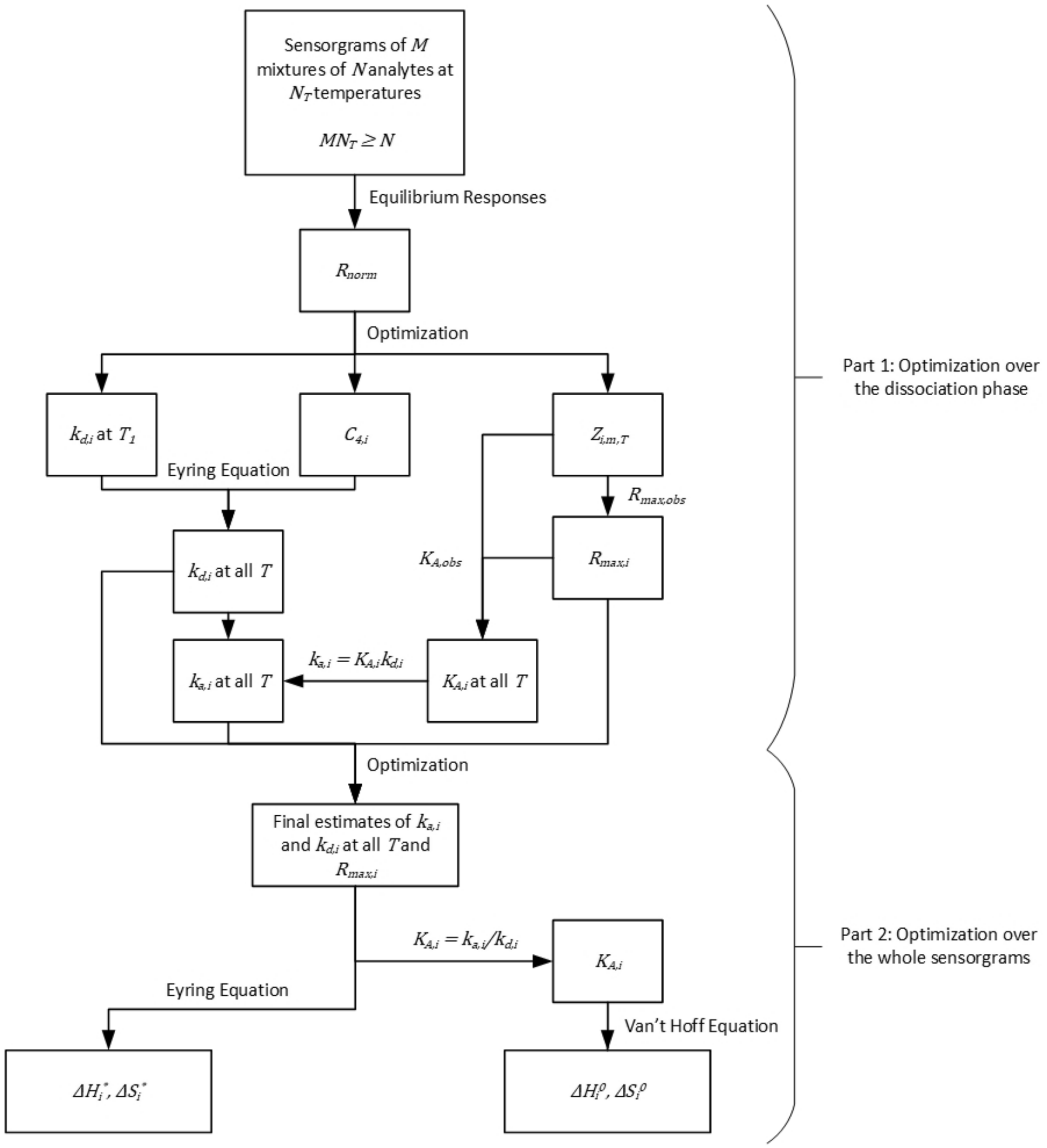
Algorithm for the estimation of the kinetic parameters of $N$ analytes at ${N}_{T}$ temperatures. The first part of the algorithm surveys the dissociation phase of the sensorgrams. The results of the first part are used as a starting point for the second part which consists of a second optimization routine over the whole sensorgrams that leads to the final estimates. Estimates of ${k}_{a,i,T}$, ${k}_{d,i,T}$ and ${R}_{max,i}$ for $i=1, \dots , N$ and $T=1,\dots ,{N}_{T}$ can be obtained from SPR sensorgrams of $M$ mixtures of the $N$ analytes at ${N}_{T}$ temperatures $(M{N}_{T}\ge N)$. The fit is said 'global-global-global' as sensorgrams corresponding to different overall concentrations, different mixtures and different temperatures are used simultaneously in the fitting procedure. Thermodynamic parameters can be extracted from the kinetic parameters at different temperatures via the Van’t Hoff and Eyring equations.

The suggested analysis framework consists of a two-part algorithm that is illustrated in Fig. 1. The first part of the algorithm focuses on the dissociation phase of the recorded SPR sensorgrams. Parameters estimated in the first part will be used in the second part as a starting point for a subsequent optimization routine which takes the whole sensorgrams into account. As sensorgrams corresponding to multiple concentrations of multiple mixtures at multiple temperatures will be considered in the same fitting procedure, the fit may be called ‘global-global-global’ as opposed to ‘global’ fitting (multiple concentrations, traditional) and to ‘global-global’ fitting (multiple concentrations and mixtures^15^). As the multi-analyte SPR model is only locally identifiable^32^, providing an appropriate starting point (first part of the algorithm) is crucial to ensure convergence to a biologically meaningful solution during the second part of the algorithm, hence the pertinence of the present work.

#### First part: optimization over the dissociation phase

##### Parameters to identify

The parameter vector $\theta$ contains a total of $N\cdot ({N}_{T}+2)$ parameters:

- The equilibrium contribution of each analyte in the first mixture (reference mixture ${m}_{1}$) (${Z}_{i,{m}_{1},T}$) for each temperature ($N\cdot {N}_{T}$ parameters).
- The dissociation rate of each analyte at the first temperature (reference temperature ${T}_{1}$) (${k}_{d,i,{T}_{1}}$, $N$ parameters).
- Temperature dependence of the dissociation rates (${C}_{4,i}$, $N$ parameters).

##### Required data

To identify the parameter vector $\theta$, the following conditions on the available data must be met:

- Minimally $$M\ge 2$$ mixtures
  o $M<N$, otherwise the method described in^15^ may be applied independently for each temperature.
  o The condition $\frac{{F}_{i,{m}_{1}}}{{F}_{i,{m}_{2}}}\ne \frac{{F}_{j,{m}_{1}}}{{F}_{j,{m}_{2}}}$ must be verified for each analyte couple $\left(i,j\right)$ to validate structural identifiability i.e., the analytes fractions in different mixtures must not be perfectly correlated.
- ${N}_{T}$ injection temperatures such that ${N}_{T}\cdot M\ge N$.

##### Objective function

The optimization routine aims to minimize the sum of squared residuals between the predicted normalized response and the observed normalized response. This is done for each analyte concentration, mixture and temperature, hence ‘global-global-global’ fitting.

For mixture $m$ injected at temperature $T$, the normalized response (independent of concentration) can be expressed in this manner (from (11), (12) and (14)):

15 $${R}_{norm,m,T}^{pred}\left(t\right)=\sum_{i=1}^{N}{Z}_{i,{m}_{1},T}\frac{{F}_{i,m}}{{F}_{i,{m}_{1}}}\frac{{\left({R}_{max,obs}{K}_{A,obs}\right)}_{{m}_{1},T}}{{\left({R}_{max,obs}{K}_{A,obs}\right)}_{m,T}}\mathrm{exp}\left(-{k}_{d,i,{T}_{1}}\frac{T}{{T}_{1}}\mathrm{exp}\left({C}_{4,i}\left(\frac{1}{T}-\frac{1}{{T}_{1}}\right)\right)t\right)$$

The observed normalized response is obtained directly from the recorded sensorgrams (see (14)):

16 $${R}_{norm,m,T}^{obs}(t)=\frac{{R}_{tot,m,T}^{obs}\left(t\right)}{{R}_{tot,m,T}^{obs}({t}_{diss})}$$

Finally, the value of the objective function $J$ is given by:

17 $$J\left(\theta \right)=\sum_{T}\sum_{M}\sum_{t}{\left({R}_{norm,m,T}^{pred}\left(t\right)-{R}_{norm,m,T}^{obs}\left(t\right)\right)}^{2}$$

As ${R}_{norm}$ is not dependent on concentration, normalized responses obtained for different concentrations may be averaged to obtain ${R}_{norm}^{obs}\left(t\right)$.

Various boundaries and constraints are added to the problem. These ensure convergence to a solution that is biologically meaningful. For example, no analyte may cause a negative response, and kinetic rates should be positive and only increase with temperature. Those are discussed in detail in Supplementary Materials, as well as a relevant starting point for the optimization routine.

##### Identification of the maximum response amplitude for each analyte

After the optimization problem has been solved, we have access to $Z_{{i,m,T}}$ for all analytes for each mixture and temperature. From the definition of the equilibrium contribution (10), we have:

18 $$\frac{{Z}_{i,m,T}}{{R}_{max,i}}=\frac{{F}_{i,m}{K}_{A,i,T}}{{\left({K}_{A,obs}{R}_{max,obs}\right)}_{m,T}} \forall m,T$$

From the definition of the observed affinity (8), we can sum on the analytes on both sides to obtain:

19 $$\begin{aligned}\sum_{i=1}^{N}\frac{{Z}_{i,m,T}}{{R}_{max,i}}&=\sum_{i=1}^{N}\frac{{F}_{i,m}{K}_{A,i,T}}{{\left({K}_{A,obs}{R}_{max,obs}\right)}_{m,T}} \forall m,T\\ \sum_{i=1}^{N}\frac{{Z}_{i,m,T}}{{R}_{max,i}}&=\frac{{\left({K}_{A,obs}\right)}_{m,T}}{{\left({K}_{A,obs}{R}_{max,obs}\right)}_{m,T}} \forall m,T\\ \sum_{i=1}^{N}\frac{{Z}_{i,m,T}}{{R}_{max,i}}&=\frac{1}{{\left({R}_{max,obs}\right)}_{m,T}} \forall m,T \end{aligned}$$

We obtain a $Ax=b$ type linear system of equation with ${N}_{T}\cdot M$ equations and $N$ unknowns that can be solved to obtain $\frac{1}{{R}_{max,i}}$ for each analyte which is inverted to obtain ${R}_{max,i}$.

The resolution of this system requires to choose the number of temperatures at which the experiments are performed such that ${N}_{T}\cdot M\ge N$. Comparing with previous work^15^, the information that is missing because $M<N$ is compensated by surveying different temperatures.

##### Affinity of each analyte at each temperature

Having access to ${Z}_{i,{m}_{1},T}$ and the ${R}_{max,i}$, we obtain the affinity of each analyte at each temperature from the definition of the equilibrium contributions (10):

20 $${K}_{A,i,T}=\frac{{Z}_{i,{m}_{1},T}{\left({K}_{A,obs}{R}_{max,obs}\right)}_{{m}_{1},T}}{{F}_{i,{m}_{1}}{R}_{max,i}} \forall i,T$$

##### Association rate of each analyte at each temperature

Because the ${k}_{d,{i,T}_{1}}$ and ${C}_{4,i}$ are now known, the dissociation rate of each analyte can be calculated for any $T$. By definition of the affinity, which was just obtained, we have:

21 $${k}_{a,i,T}={K}_{A,i,T}{k}_{d,i,T}$$

#### Second part: optimization over the whole sensorgrams

##### Final estimates

To obtain the final estimates of the kinetic constants and response amplitudes, we feed the results of the first part of the algorithm to a second optimization routine, which takes the whole sensorgrams into account, rather than only the dissociation phase. Again, sensorgrams obtained from different mixtures injected at different concentrations and temperatures will be considered, hence we perform a ‘global-global-global’ fit. $N\cdot \left(2{N}_{T}+1\right)$ parameters need to be identified here. Constraints may be added to ensure that kinetic rates increase with temperature. Because an appropriate starting point is provided, convergence to a biologically sensible solution should be attained. This is not necessarily the case for any starting point, as the multi-analyte SPR model is not globally structurally identifiable^32^.

At this stage, one can add local bulk effect contribution parameters (${R}_{I}$, one per sensorgram) to account for the imperfect referencing of the buffer change effect during the association phase. Hence, the predicted sensorgram becomes:

22 $${R}_{TOT}=\left\{\begin{array}{l}\sum_{i=1}^{N}{R}_{i}+{R}_{I}\;injection\;phase\\ \sum_{i=1}^{N}{R}_{i}\; dissociation\;phase\end{array}\right.$$

##### Thermodynamic parameters estimates

Having access to ${k}_{a,i}$, ${k}_{d,i}$ and ${K}_{A,i}$ at multiple temperatures, the parameters of the Eyring and Van’t Hoff equations are directly obtained via a simple linear regression. This enables the calculation of these parameters for any temperature. The Eyring equation is given in (4) and (5). The Van’t Hoff equation is given by:

23 $$\mathrm{ln}\left({K}_{A}\right)=-\frac{\Delta {H}^{0}}{{R}_{IG}T}+\frac{\Delta {S}^{0}}{{R}_{IG}}$$

where $\Delta {H}^{0}$ and $\Delta {S}^{0}$ are respectively the standard reaction enthalpy and entropy of the analyte-ligand complex formation pseudo-reaction.

### Composition estimation

Having determined the kinetic and thermodynamic parameters of the analytes, our second algorithm aims to use that information to estimate the composition of an unknown mixture of the analytes. As an extension of previous work by our group^15^, we will now consider the case where the concentration of the mixture is also unknown. However, we will consider that the dilution factors used to generate dilutions of a stock solution of the mixture are known.

#### Composition estimation with unknown total concentration

**Figure 2:**
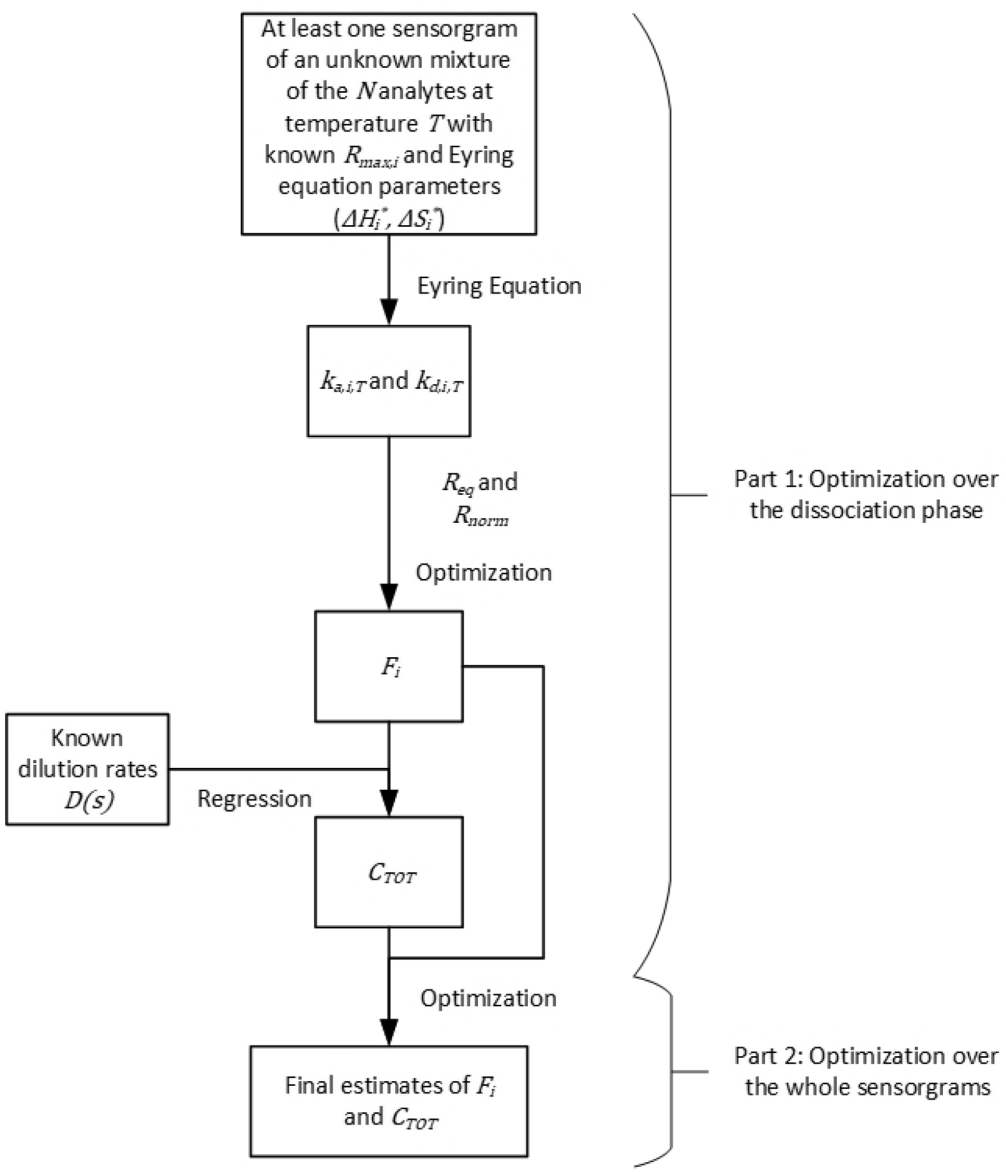
A two-part algorithm for the estimation of the composition and concentration of an unknown mixture of $N$ analytes with known thermodynamic parameters. The unknown mixture is injected at temperature $T$ for which corresponding kinetic parameters may be calculated using the Eyring equation. The first part of the algorithm surveys the dissociation phase of the sensorgrams to provide a first estimate of the fractions ${F}_{i}$ of each analyte. The starting point for this optimization routine is set to $1/N$. A first estimate of the total concentration ${C}_{TOT}$ can then be found via linear regression. The second part of the algorithm considers the whole sensorgrams to provide a final estimate of ${F}_{i}$ and ${C}_{TOT}$. Note that, if sensorgrams of more than one total concentration are available, the corresponding dilution rates ($D\left(s\right)$) must be known.

Similarly to the parameter identification algorithm, the proposed algorithm for composition estimation also has two parts. The first one focuses on the dissociation phase of the SPR sensorgrams. The method can theoretically be used with only one sensorgram of the unknown mixture at one temperature, as long as an equilibrium plateau is reached during the association phase. Figure 2 illustrates the algorithm.

##### First part: optimization over the dissociation phase

From the definition of the normalized SPR response during the dissociation phase with respect to the equilibrium contributions given in (14), we aim to minimize the following objective function via optimization:

24 $$J\left(\theta \right)=\sum_{t}{\left({R}_{norm}^{pred}\left(t\right)-{R}_{norm}^{obs}\left(t\right)\right)}^{2}$$

Here, the vector of identified parameters $\theta$ contains the fraction of each analyte in the unknown mixture ${F}_{i}$. Given a set of ${F}_{i}$, the contributions ${Z}_{i}$ can be calculated. Constraints should be added such that ${F}_{i}$ are contained between zero and one, and that they sum to one. The starting point for each ${F}_{i}$ can be set to $1/N$.

Having identified the fractions, the observed affinity and maximum response can be obtained from (8) and (9). We can rearrange the expression of the equilibrium response given in (6) to obtain:

25 $$\frac{{R}_{max,obs}{K}_{A,obs}}{{R}_{eq}\left(s\right)}-{K}_{A,obs}=\frac{1}{D\left(s\right)}\cdot \frac{1}{{C}_{TOT,REF}}$$

Here $D\left(s\right)$ is the vector of dilution factors used to obtain the analyte concentration injected to obtain sensorgram $s$. If ${C}_{TOT,REF}$ is the concentration of the initial solution that was used to create a dilution series, we have ${C}_{TOT}\left(s\right)=D\left(s\right){{C}_{TOT}}_{REF}$. At this stage, everything is known in (25) except ${C}_{TOT,REF}$. It can be obtained directly if only one sensorgram is available, or via a simple regression with null intercept if multiple dilutions of the unknown mixture were injected.

#### Second part: optimization over the whole sensorgrams

The second part of the algorithm consists in using the ${F}_{i}$ identified from the dissociation phase and the ${C}_{TOT,REF}$ identified from the equilibrium values as the starting point of a second optimization routine that considers the entire sensorgrams (injection and dissociation phases). The same constraints are applied on the ${F}_{i}$, and the objective function is:

26 $$J\left(\theta \right)=\sum_{s}\sum_{t}{\left({R}^{pred}\left(t\right)-{R}^{obs}\left(t\right)\right)}^{2}$$

This leads to the final estimates of ${F}_{i}$ and ${C}_{TOT,REF}$, from which the analyte concentration of all sensorgrams can be computed from the known $D\left(s\right)$. Bulk effect contribution parameters may be added for each sensorgram as additional identified parameters in optimization problem (26).

## Results

### Parameter identification

To test our parameter identification algorithm, we immobilized CAII on SPR biosensor surfaces. It has been confirmed by SPR studies many times that CAII binds several small compounds following a simple 1:1 Langmuir scheme^10–12,15,28–30^. We selected four compounds amongst the known CAII binders: CBS, BDS, sulfanilamide and furosemide and we created four mixtures containing all of them (Table 1). For details on the binding mechanisms of sulfonamides to CAII, we refer to a literature review^33^. These binders were selected because they exhibit a wide range of kinetic and thermodynamic parameters and refractive index increments (RII). To account for the differences in RII, our model considers distinct maximum response amplitudes (${R}_{max,i}$) for each analyte. The model also assumes that there is competition between the analytes (binders) and that each analyte binds CAII following a 1:1 scheme^10,12,15^.

To push the boundaries of pre-existing work, where four mixtures were needed to elucidate the kinetics of four analytes ($M=N$), we proposed to reduce the demand in mixtures ($M<N$) by performing experiments at different temperatures. Hence, each of the mixtures was injected at four temperatures and seven dilutions. In addition, classical experiments where the analytes were injected alone were also performed at each temperature and for each analyte. The recorded sensorgrams allowed us to identify kinetic parameters at all four temperatures, while enthalpies and entropies were derived using the Eyring and Van’t Hoff equations. All the calculated parameters are reported in Supplementary Materials Tables S1 and S2. The standard errors we computed for parameters fitted via single-analyte experiments corresponded to less than 1% of the fitted parameter in all cases and were in good agreement with those obtained with the Biacore Evaluation Software^31^.

**Figure 3:**
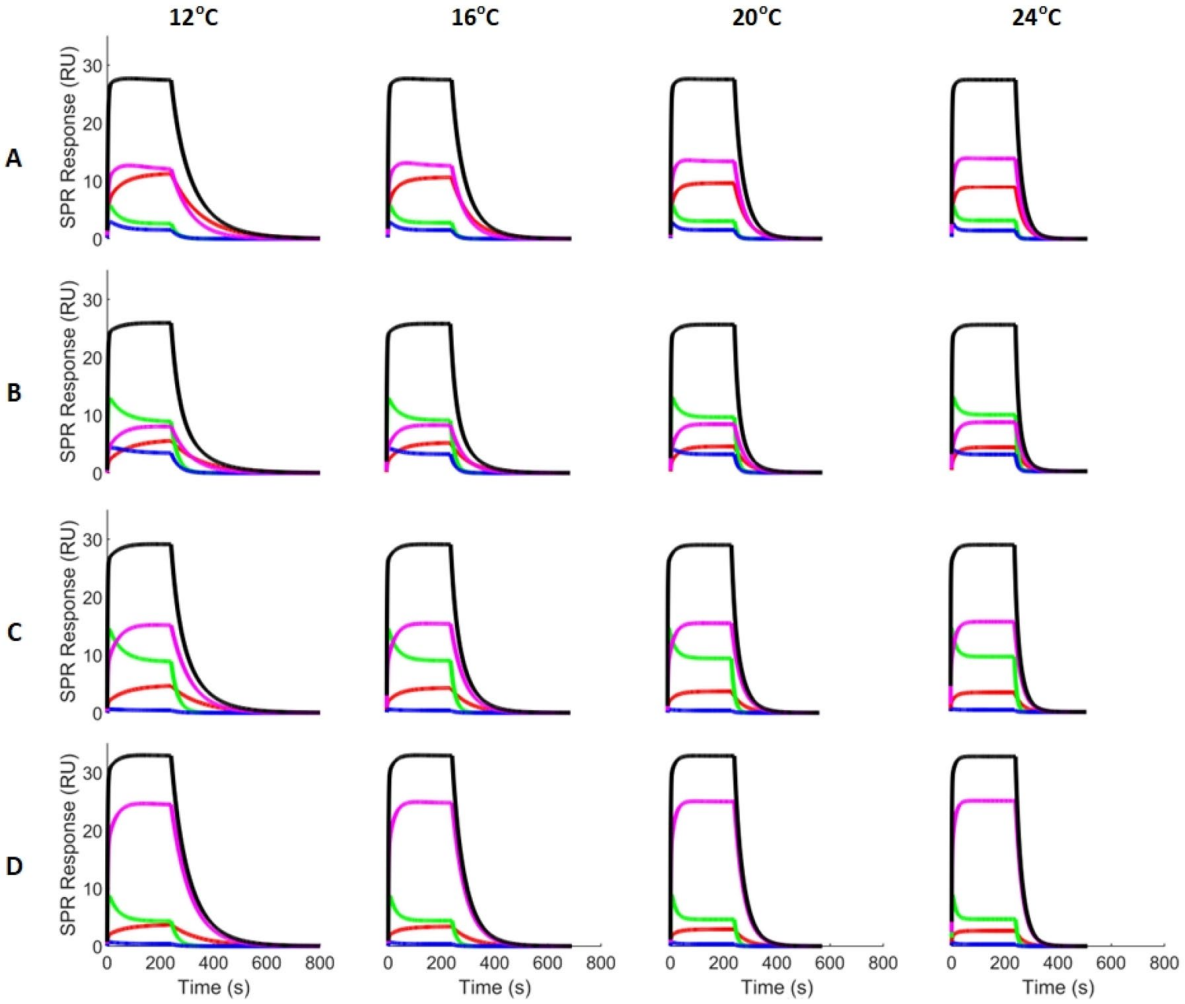
Kinetic analysis of the contribution of CBS (red), BDS (green), sulfanilamide (blue) and furosemide (magenta) to the SPR response of each mixture (see Table 1) at each temperature. The kinetic parameters identified with classical single-analyte experiments were used to generate this figure. The summation of these signals gives the predicted signal (black). Only the responses corresponding to the maximal concentration (22 400 nM) are shown.

We used the kinetic parameters identified with classical single-analyte experiments to evaluate the actual contribution of each analyte for each mixture at each temperature (see Fig. 3). This can be done by solving the ODEs given in (3) for each ${R}_{i}$. Of interest, BDS is seen to rapidly bind to CAII, which is indicative of a high association rate. However, in every case, it is progressively replaced either by a slower binder with a higher affinity (i.e., furosemide) or by an analyte with a higher proportion in the mixture. CBS and furosemide exhibit the slowest dissociation (lowest ${k}_{d,i}$) while sulfanilamide and BDS rapidly dissociate from CAII. Furosemide has the highest maximal response (${R}_{max,i}$) and hence it always has a significant contribution. On the other hand, sulfanilamide has the lowest affinity for CAII amongst the studied binders (by almost one order of magnitude). This explains why its contribution is always modest to negligible, even in mixture B where it is present in a proportion of 50%. These observations will be relevant when analyzing the performance of our parameter identification algorithm.

We first applied our parameter identification algorithm to all four combinations of three mixtures. To evaluate the loss of performance when less data is available, we repeated the procedure with all the six combinations of two mixtures. As sensorgrams corresponding to different concentrations, mixtures and temperatures are simultaneously used to fit the model, this procedure may be called ‘global-global-global’. Parameters identified with our suggested algorithm were compared to those obtained by performing classical single-analyte experiments via what we call the deviation:

27 $${\%deviation}_{k}=\frac{\left|{k}_{pred}-{k}_{1 analyte}\right|}{{k}_{1 analyte}}*100$$

where $k$ denotes any fitted parameter and ${k}_{1 analyte}$ is the value of the parameter as derived through a single-analyte experiment.

#### Parameter identification with three mixtures

Kinetic parameters derived by fitting the multi-analyte model to sensorgrams of three mixtures at four temperatures are reported in Supplementary Materials Table S1. In all cases, the reference temperature ${T}_{1}$ was 12 °C and the reference mixture ${m}_{1}$ was the first mixture in the data set name (for part 1 of the algorithm, see Eq. (15)). 95% confidence intervals were also calculated (see Supplementary Materials). The affinity ${K}_{A}$ is not directly fitted by the algorithm but is rather derived from the association and dissociation rates. Hence, its confidence interval was calculated by propagating the standard error of ${k}_{a}$ and ${k}_{d}$. In all cases, the amplitude of the confidence interval corresponds to less than 7% of the fitted parameter value, with association rates having the widest intervals. Association rates tend to be more impacted by experimental error, as they always multiply the analyte concentration in the model ODEs, which is dependent on the precision of the experimenter. The quality of the fits is excellent, as can be assessed by the low χ^2^ values corresponding to all multi-analyte fits (${\chi }^{2}=\sim 0.024$ for all fits). The square root of χ^2^ corresponds to the root mean square error and should approximate the measurement noise, if the model is appropriate. Some constraints on the association rate of sulfanilamide were active in some of the fits. These constraints ensure that the kinetic rates only increase with respect to temperature. However, active constraints obscure the meaningfulness of the calculated confidence intervals of the affected parameters as well as that of the thermodynamic parameters derived from those kinetics.

**Figure 4:**
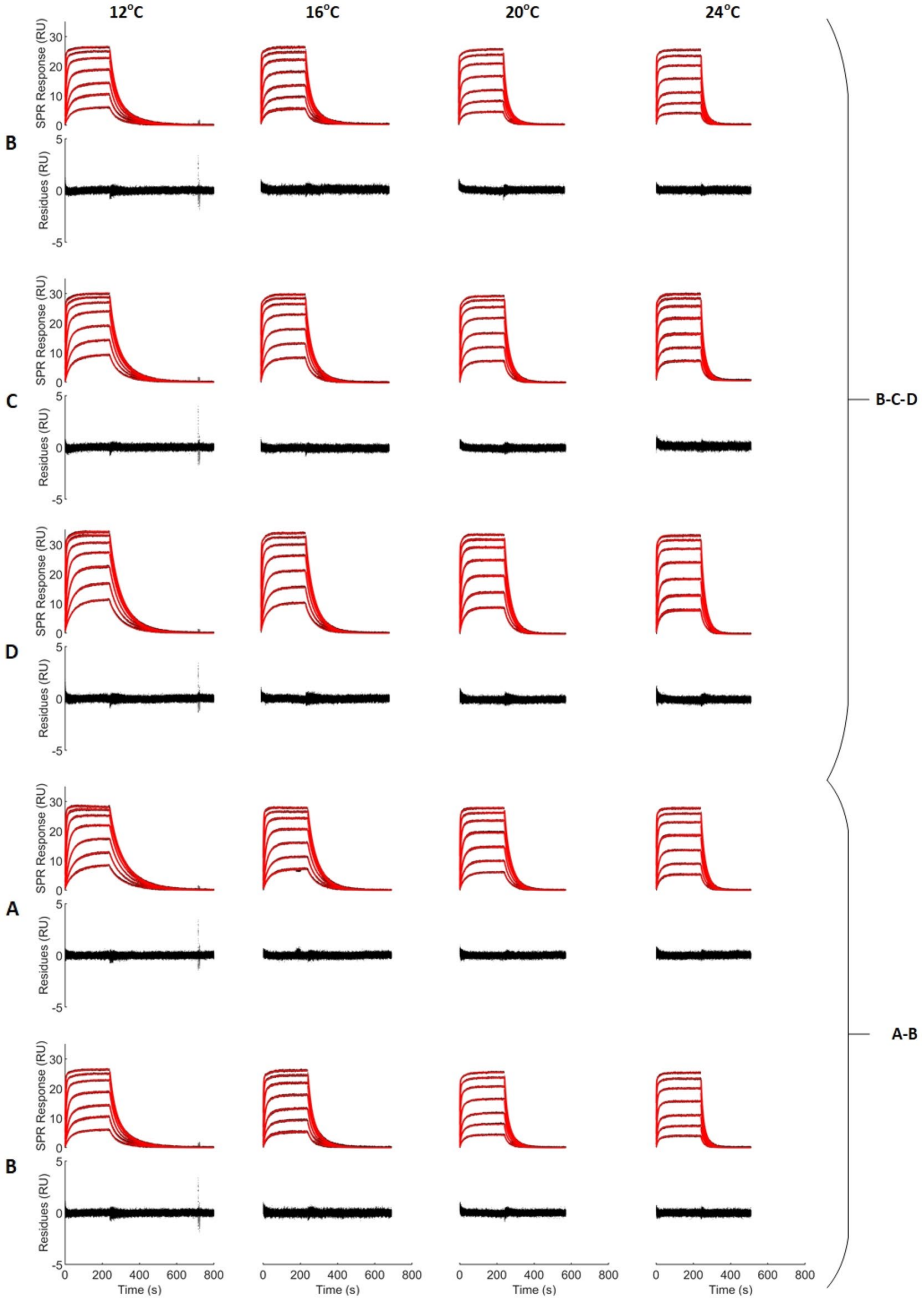
Kinetics analysis of the injection of mixtures of four compounds at four temperatures. Black dots correspond to control-corrected and double-referenced sensorgrams. Red lines correspond to ‘global-global-global’ fits for each data set (B–C–D and A–B, see Table 1 for exact mixture compositions). The total concentration (${C}_{TOT}$) of the mixtures ranged from 350 nM to 22,400 nM for all mixtures and temperatures. Here, the composition and total concentration are assumed to be known. The related residual plot is given below each sensorgram data set.

We show an example of a multi-analyte fit to mixtures B-C-D in Fig. 4. A residue analysis shows no obvious trend and very small residues, hence the 1:1 competitive multi-analyte model adequately represents the data, as expected for this system. During fitting, the first and last second of the injection phase and the first second of the dissociation phase were removed to avoid artifacts hailing from buffer changes between the different phases. In 12 °C sensorgrams, a spike is always observed around 700 s. This occurs when the buffer syringe needs to be refilled due to the longer dissociation phase at this temperature.

**Figure 5:**
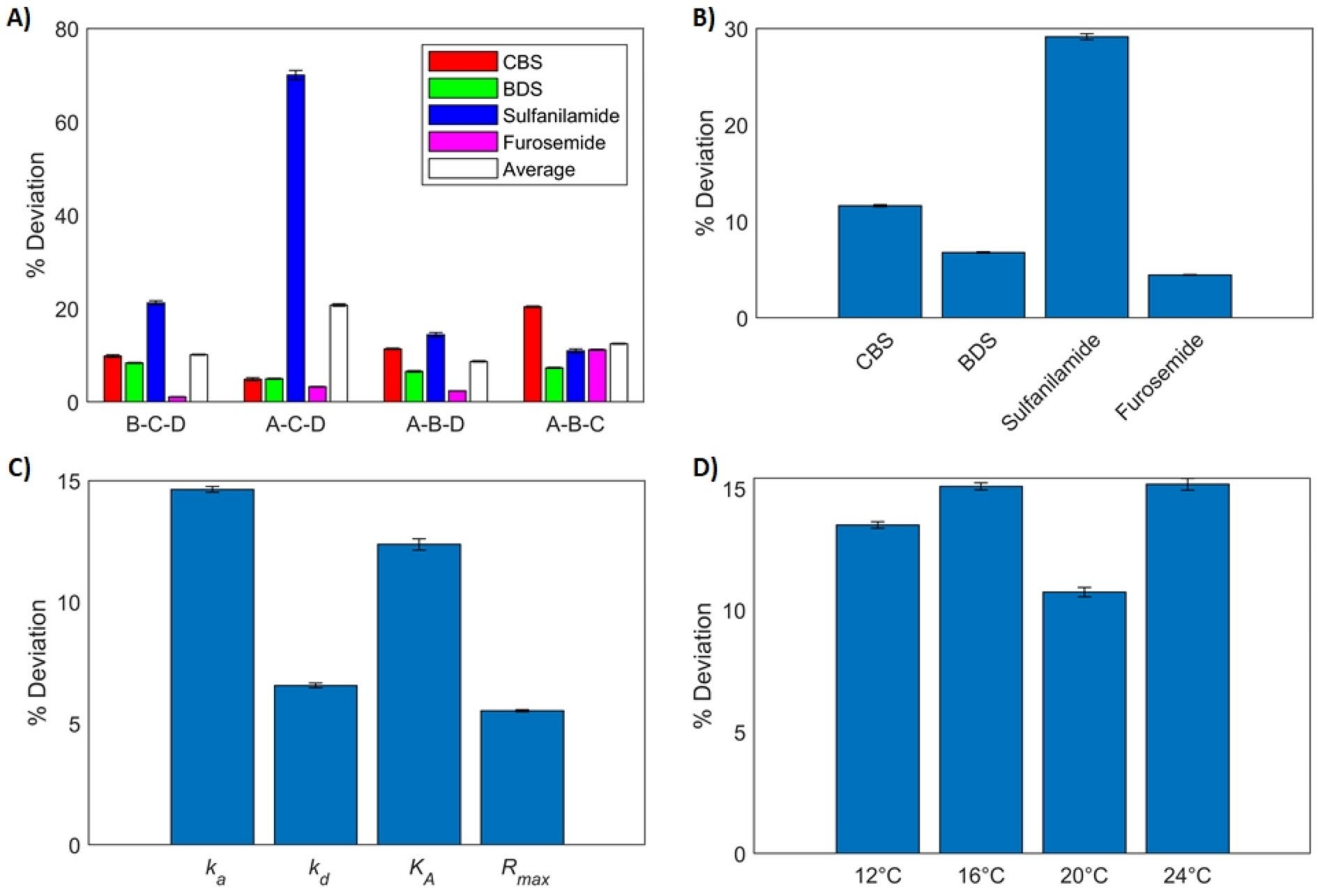
Average deviation between the parameters (${k}_{a}$, ${k}_{d}$, ${K}_{A}$ and ${R}_{max}$) identified from single-analyte experiments and parameters identified by our multi-analyte model with any combination of three mixtures (out of the four mixtures described in Table 1). In all cases, data generated at four temperatures were taken into account. (**A**) Average deviation computed over all parameters and temperatures for all analytes in all fits. (**B**) Average deviation over all parameters, fits and temperatures for each analyte. (**C**) Average deviation for all parameters. (**D**) Average deviation for all temperatures. Error bars were computed by propagating the standard error of the parameters identified from single-analyte and multi-analyte experiments.

Figure 5 reports the deviation (in %) between the kinetic parameters derived from single- and multi-analyte experiments. All fits showed similarly good accuracy with deviations on the order of 10%, except for that of data set A–C–D (Fig. 5A). For this data set, estimates of the parameters of sulfanilamide had a deviation of approximately 70% on average. As observed earlier (Fig. 3), sulfanilamide had a negligible contribution to the SPR response for mixtures C and D, and a very modest one in mixture A. That is, the predicted SPR response had a weak dependence on the parameters of this analyte. Hence, the quality of the fit depended weakly on the sulfanilamide parameters, making them difficult to identify accurately using this data set. This phenomenon is avoided with all other data set thanks to the presence of mixture B, where sulfanilamide is present in a large proportion and contributed more appreciably to the SPR response. Even with the other data sets, deviations on the parameters of sulfanilamide remained higher on average (Fig. 5B). Furosemide is the analyte with the highest affinity for CAII, and thus the highest contribution to the SPR response. As such, it was also the analyte for which the kinetic parameters are the most accurately identified with less than 5% deviations on average. The association rates were the parameters with the highest average deviation (Fig. 5C). As previously mentioned, this is because they always multiply the concentration (and fraction) in the ODEs, which is dependent on the experimenter’s repeatability when creating the mixtures and performing the necessary dilutions from one experiment to another. Moreover, the association rates only directly affect the injection phase of the sensorgrams. On the contrary, the ${R}_{max,i}$ were most precisely identified. As these are considered temperature invariant, they have a stronger impact on the quality of the fit than any single-temperature association or dissociation rate. Hence, the fit should be more sensitive to the ${R}_{max,i}$. Dissociation rates were also accurately identified, as they affect both phases of the sensorgrams. Affinities were obtained by computing the ratio of the association and dissociation rates rather than being directly identified, hence the precision of the predicted affinities is dependent on the precision of the predicted kinetic rates. Finally, kinetic parameters corresponding to all temperatures were identified with a similar accuracy (Fig. 5D).

Having derived the association and dissociation rates, and the affinity at four different temperatures, we identified the Eyring and Van’t Hoff equation parameters via linear regression. Table S2 in Supplementary Materials reports the activation and standard reaction enthalpies and entropies of the analyte-ligand complex formation pseudo-reaction. Considering the standard Gibbs energy $\Delta {G}^{0}=\Delta {H}^{0}-T\Delta {S}^{0}$, all analyte complex formations were favored by enthalpic contributions (negative enthalpy change), whereas CAII binding to BDS or furosemide was also supported by entropic contributions (positive entropy change).

**Figure 6:**
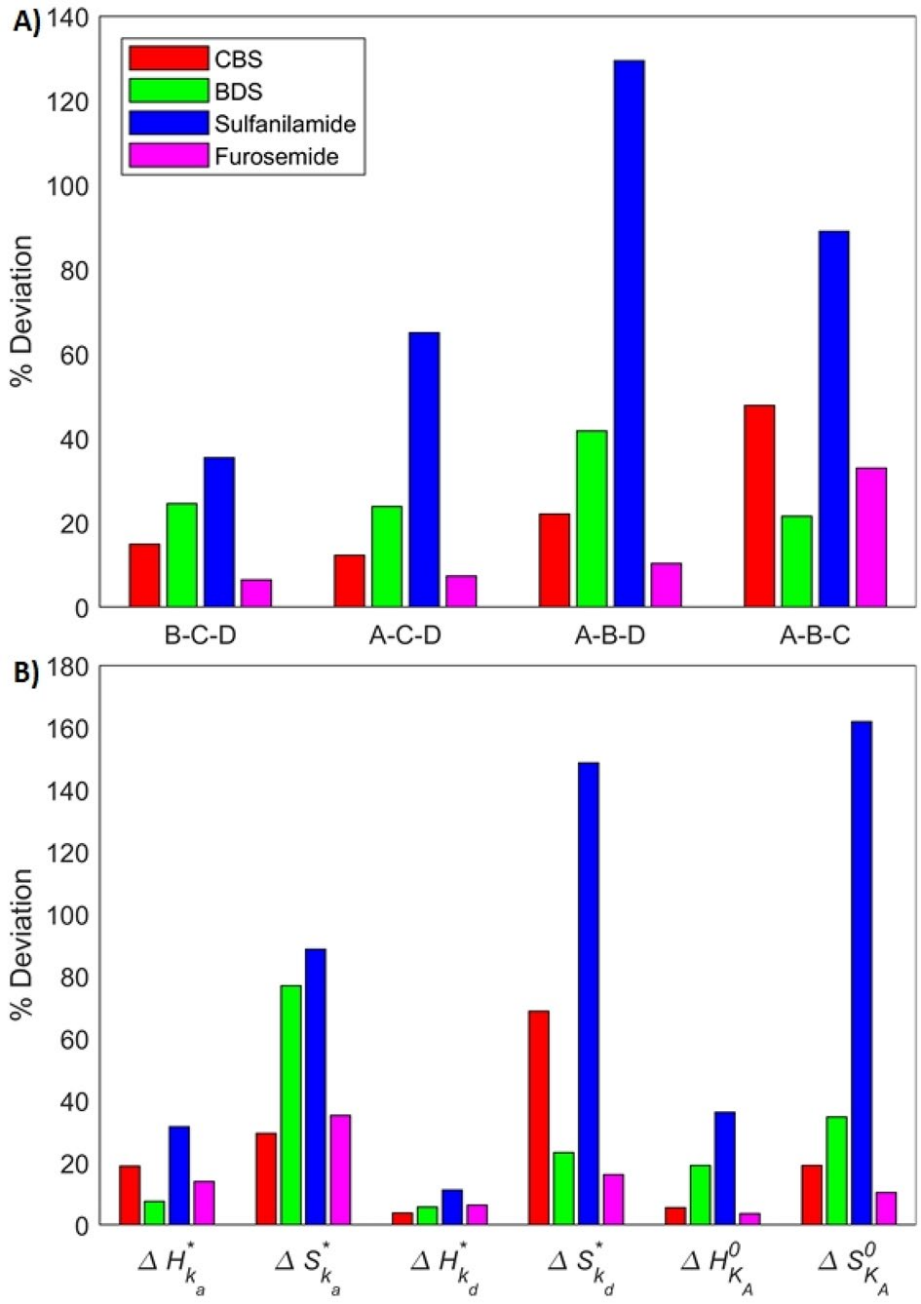
Average deviation between the thermodynamic parameters ($\Delta {H}_{{k}_{a}}^{*}$, $\Delta {S}_{{k}_{a}}^{*}$, $\Delta {H}_{{k}_{d}}^{*}$, $\Delta {S}_{{k}_{d}}^{*}$, $\Delta {H}_{{K}_{A}}^{0}$ and $\Delta {S}_{{K}_{A}}^{0}$) derived from single-analyte experiments and parameters derived by fitting the multi-analyte model at four temperatures with any combination of three mixtures (**A**–**D**). (**A**) Average deviation computed over all parameters for all analytes in all fits. (**B**) Average deviation computed over all fits for all parameters of all analytes.

Figure 6 compares the thermodynamic parameters derived from multi-analyte experiments to those derived from single-analyte experiments. The thermodynamic parameters of sulfanilamide exhibit the biggest deviations in all fits, as expected because of its low contribution to the SPR response (Fig. 6A). Data set B–C–D produced a fit with no active constraint, leading to the most precise identification of the thermodynamic parameters (35% deviation for sulfanilamide and below 25% for all other analytes). On the other hand, fit A-B-D had the largest number of active constraints, leading to the largest deviations. The constraints that are active are those that ensure that the association rates only increase with temperature. This in turn affects the computation of the corresponding enthalpic and entropic changes.

Enthalpies were accurately identified (average deviation of ~ 13% between single- and multi-analyte experiments averaged over Eyring and Van’t Hoff parameters found with all data sets) whereas entropy estimates were less accurate (average deviation of ~ 59%), as is shown in Fig. 6B. The enthalpies modulate the temperature dependence of the kinetic parameters (or affinity) while the entropies represent the base level, which may be more sensitive to experimental error during sample dilution. All predicted enthalpies and entropies had the correct sign except for entropies associated with the dissociation rate ($\Delta {S}_{{k}_{d}}^{*}$), mainly for sulfanilamide. This is presumably because the absolute value of this entropy seems to be close to 0 for sulfanilamide. Our algorithm can provide at least coarse first estimates when analyzing a mixture of analytes that is difficult to purify, but likely cannot compete with specialized methods, like calorimetry, in accuracy when pure samples are available.

#### Importance of the initial estimates for parameter identification

Part 1 of our algorithm favors convergence to a proper, biologically relevant solution when performing part 2. This is necessary because the multi-analyte model is only locally identifiable^32^. To demonstrate this point, we performed the optimization routine of part 2 of our algorithm with a suboptimal starting point for data set B–C–D. The suboptimal starting point was constructed by assigning the value of the expected order of magnitude of each parameter (10^4^ s^−1^ M^−1^ for ${k}_{a}$, 10^–2^ s^−1^ for ${k}_{d}$ and 10 for ${R}_{max}$). With this data set, the optimizer converged to a suboptimal solution (χ^2^ of 0.0301 vs 0.0247) that was further from single-analyte estimates as the one obtained with our algorithm (average deviation over all analytes, parameters and temperatures of 74% vs 10%). Moreover, convergence necessitated approximately three times as many iterations when using a suboptimal starting point. The fitted parameters obtained with a suboptimal starting point are given in Supplementary Materials Table S3.

#### Parameter identification with two mixtures

Parameters identified by fitting the multi-analyte model at four temperatures for any combination of two mixtures (instead of three, as in the above sections) are reported in Supplementary Materials Table S4. Confidence interval lengths and χ^2^ values were similar to those obtained when using three mixtures. An example of fit is shown in Fig. 4 for data set A–B. Once again, no obvious trend was observed in the residues and the model adequately depicted the data.

**Figure 7:**
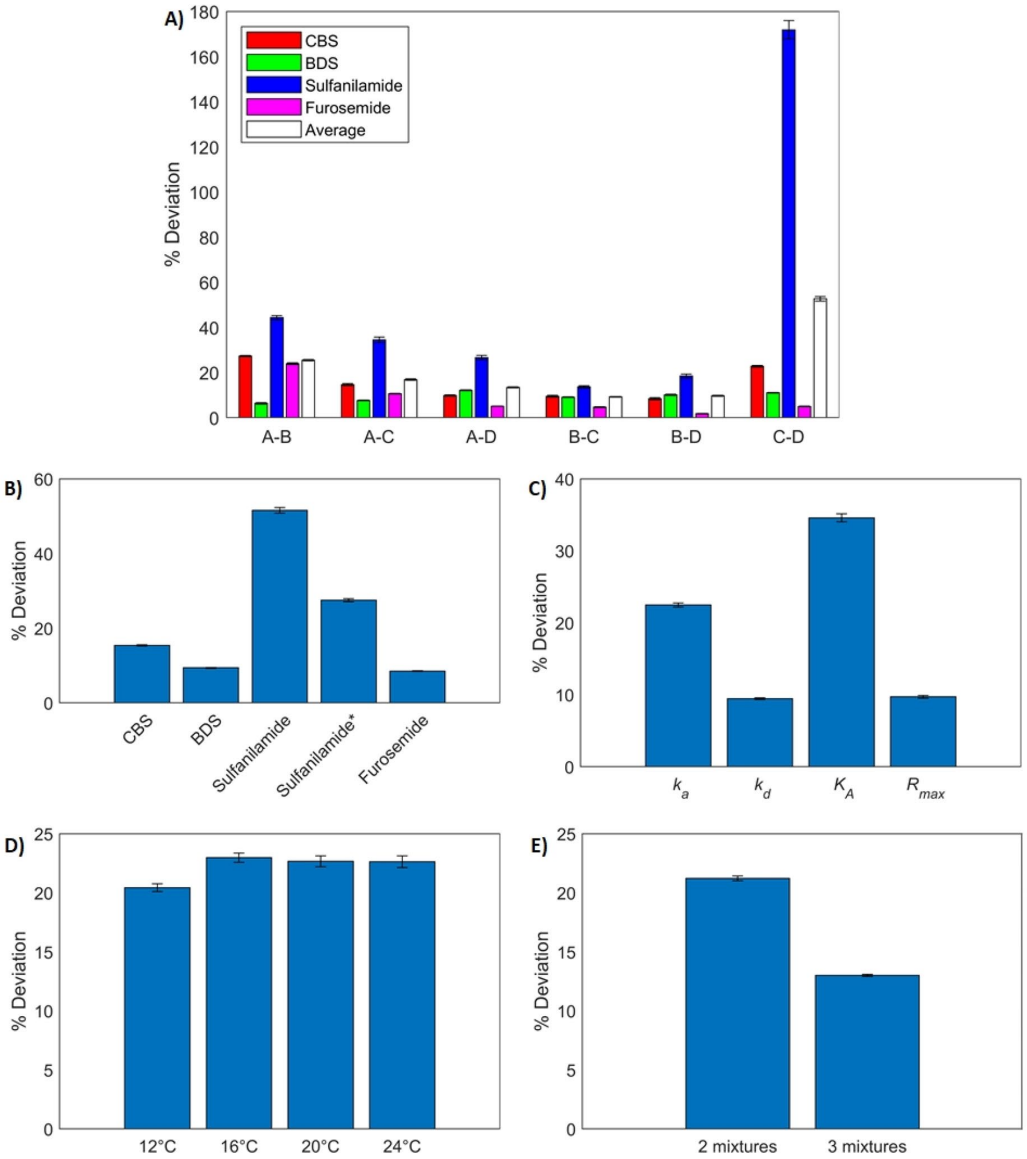
Deviation between the parameters (${k}_{a}$, ${k}_{d}$, ${K}_{A}$ and ${R}_{max}$) identified from single-analyte experiments and parameters identified by fitting the multi-analyte model at four temperatures with any combination of two mixtures (out of the four described in Table 1). (**A**) Average deviation computed over all parameters and temperatures for all analytes in all fits. (**B**) Average deviation for all analytes over all parameters, fits and temperatures. Average deviations for sulfanilamide are also reported without considering data set (C–D) (sulfanilamide*). (**C**) Average deviation for all parameters. (**D**) Average deviation for all temperatures. (**E**) Average deviation for data sets with two and three mixtures. Error bars were computed by propagating the standard error of the parameters identified from single-analyte and multi-analyte experiments.

Figure 7 shows the deviation (in %) between the kinetic parameters obtained with multi-analyte fits with two mixtures and those obtained with single-analyte experiments. A similar accuracy was obtained with all data sets except for data set C–D (Fig. 7A). With this data set, deviations on sulfanilamide parameters were over 100% on average (a more than two-fold difference from single-analyte experiments). This is mainly because sulfanilamide had a negligible contribution to the SPR response in both mixtures. Interestingly, the other three analytes were properly identified even in the C–D data set, leading to similar deviations as those obtained with three mixtures (Fig. 7B). Average deviations for sulfanilamide were larger when fitting to two mixtures compared to three (~ 50% vs ~ 30% if we exclude data set C–D). Dissociation rates and maximal responses were again the most precisely identified parameters, while association rates and affinities were more difficult to extract (Fig. 7C), as was the case for three-mixture data sets. Parameters corresponding to each temperature were identified with approximately the same precision (Fig. 7D). Overall, the parameter estimation was more precise with three-mixture data sets with an average deviation of ~ 13% than with two-mixture data sets with an average of ~ 21% (Fig. 7E).

Thermodynamic parameters derived from two-mixture data sets are shown in Table S5 in the Supplementary Materials. Again, the thermodynamic parameters of sulfanilamide differed the most from those obtained with single-analyte experiments. Except for data set C–D, the correct sign was found for the enthalpies and the average deviation (over Eyring and Van’t Hoff enthalpies found with all data sets except C–D) was ~ 18%. Entropies were identified less precisely, with an average deviation (excluding C-D) of ~ 71%. Constraints on the association rates of CBS and sulfanilamide were active in the final solution of data set C–D. There was also an active constraint on the affinity of sulfanilamide to ensure that it varies monotonously with respect to temperature. This contributed to the larger deviations observed for this data set. For more details on the deviation values, we refer the reader to Supplementary Materials Figure S1.

### Mixture composition and concentration estimation

#### Choice of injection temperature

Before injecting an unknown mixture on the biosensor surface, the injection temperature must be determined. Having access to the kinetic parameters for theoretically any temperature within the operating range of the biosensor, after applying the parameter identification algorithm, this question is nontrivial. Selecting the temperature for which the kinetic parameters of the different analytes are most different from one another would lead to bigger differences in their binding behavior, facilitating the discrimination of the respective contributions to the SPR response. As the first part of our algorithm focuses on the dissociation phase of the sensorgrams, we hypothesized that selecting the temperature which maximizes the differences in the dissociation rates should lead to better estimates. To quantify how different the dissociation rates are for a given temperature, we suggest the following temperature dependent indicator:

28 $$I\left(T\right)=\underset{i,j}{\mathrm{min}}\left(\frac{{k}_{d,i,T}}{{k}_{d,j,T}}\right) \begin{array}{c}i,j=1,\dots ,N\\ i\ne j\\ {k}_{d,i,T}>{k}_{d,j,T}\end{array}$$

The indicator $I\left(T\right)$ is equal to or greater than one. A temperature with a small $I$ means that at least two dissociation rates are close to each other, whereas a large value of the indicator signifies that the two closest dissociation rates are rather far from one another.

We tested our hypothesis via simulations. The four analytes were considered, with their Eyring parameters derived from the single-analyte experiments. 100 mixtures were constructed by randomly generating sets of fractions ${F}_{i}$. 15 equally distanced temperatures between 12 °C and 40 °C were selected. For each temperature and mixture, sensorgrams were simulated by solving the ODEs in (3) for two-fold dilutions ranging from 400 nM to 25 600 nM. Data were generated with a frequency of 10 Hz. Gaussian noise with a null mean was added on top of the simulated sensorgrams to reproduce the typical measurement noise observed on recorded sensorgrams. The choice of Gaussian distribution for the noise was validated via a quantile–quantile plot showing the quantiles of the double-referenced baseline signal of the instrument with respect to the theoretical quantile values of a normal distribution. A standard error of 0.2 was used, which is similar to that observed for said baseline. We applied our composition estimation algorithm to each mixture and each temperature individually. We then compared the estimated composition after the first and second parts of the algorithm to the composition used to generate the data. The absolute value of the difference between the estimated and actual fractions was recorded to evaluate the quality of the estimates, i.e., the mean absolute error (MAE):

29 $$MAE=\left|{F}_{pred}-{F}_{real}\right|$$

**Figure 8:**
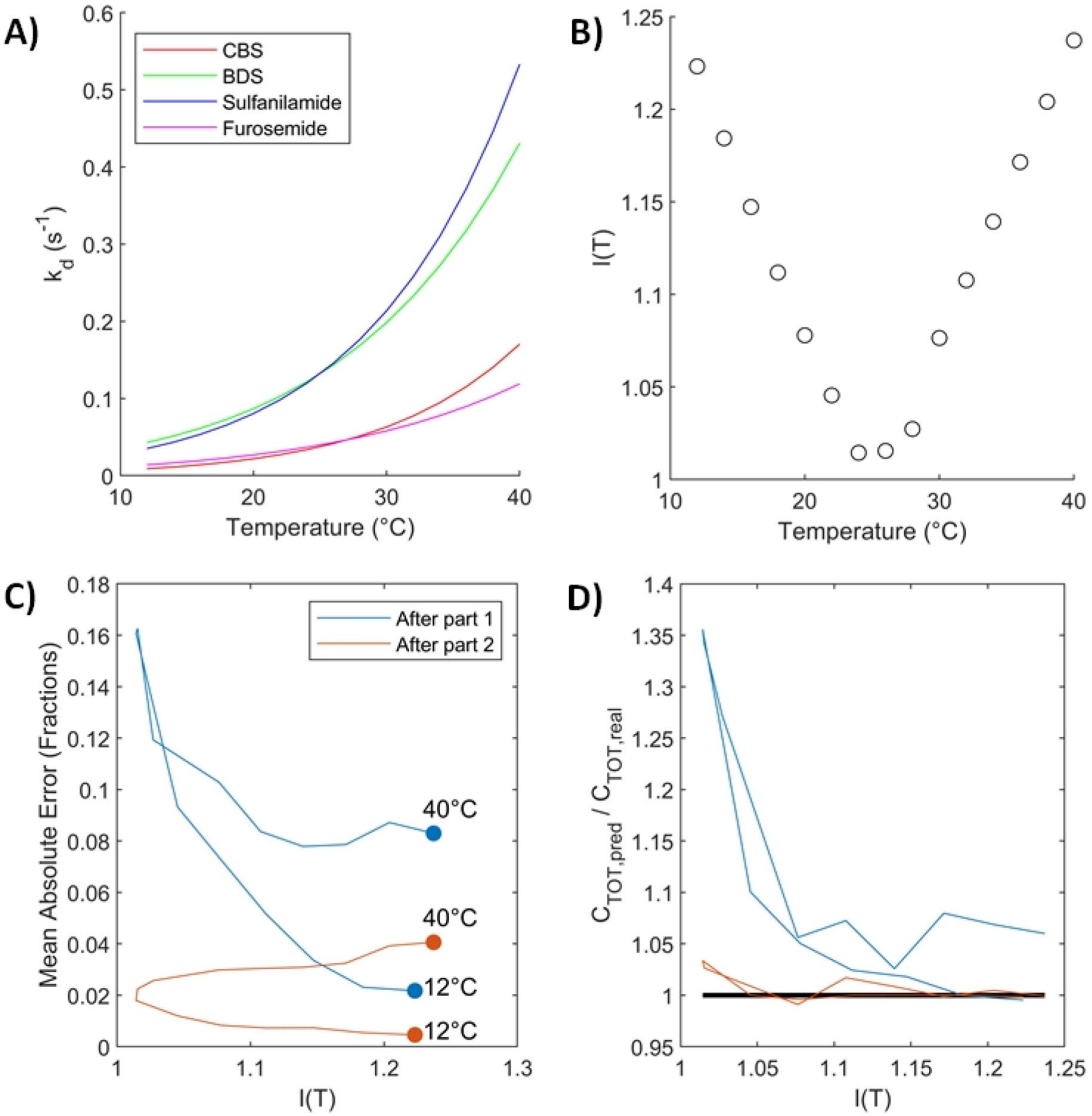
Application of the composition and concentration estimation algorithm to simulated mixtures of the four analytes at 15 temperatures between 12 and 40 °C. (**A**) Dissociation rates of each analyte with respect to temperature. (**B**) Performance indicator with respect to temperature. (**C**) Absolute values of the fraction estimation error averaged over all analytes and mixtures with respect to the indicator after part 1 and part 2 of the algorithm. To help interpretation, corresponding temperatures were added as annotations. (**D**) Ratio of estimated and actual concentrations with respect to the indicator after both parts of the algorithm. The black line corresponds to a perfect estimate (ratio of 1).

For the concentration, we recorded the ratio of the predicted value and the actual concentration: $\frac{{C}_{TOT,pred}}{{C}_{TOT,real}}$. Results of the simulations are reported in Fig. 8.

The dissociation rates of BDS and sulfanilamide and that of CBS and furosemide overlap at 25 °C and 27 °C, respectively (Fig. 8A), where the value of our suggested indicator reaches a minimum, as seen on Fig. 8B. If only part 1 of the composition estimation algorithm is performed (Fig. 8C), the absolute value of the difference between the estimated and actual fractions (averaged over the 100 simulated mixtures and 4 analytes) is negatively correlated to the indicator. That is, part 1 of the algorithm functions better when the dissociation rates are more different from one another, which was expected. There is however another factor in play, which is the injection temperature. Estimation errors tend to increase with temperature. This effect is even more noticeable in the results of part 2 of the algorithm (Fig. 8C). No obvious trend was observed when we replaced ${k}_{d}$ in the definition of $I(T)$ with any of ${k}_{a}$, ${K}_{A}$ or ${K}_{D}=1/{K}_{A}$.

The same pattern is also obtained in Fig. 8D, where the predicted concentration is compared to the actual concentration by taking their ratio. This was averaged over the 100 mixtures. The concentrations estimated by part 2 of the algorithm were within 5% of the actual fractions.

As the temperature increases, kinetics become faster and sensorgrams exhibit less curvature (more of a ‘rectangular’ shape). These ‘rectangular’ sensorgrams do not contain a sufficient amount of information to be able to discriminate between the analytes.

In general, we would advise to select the temperature that maximizes our indicator $I(T)$, while minimizing temperature, as much as possible so that equilibrium plateaus may still be reached. This will ensure part 1 of the algorithm provides an appropriate starting point for part 2, allowing faster convergence to a better estimate of the composition.

In the simulation, sulfanilamide was the analyte with the biggest estimation errors, due to its lower affinity for CAII, and hence smaller contribution to the simulated response. BDS followed, as BDS and Sulfanilamide exhibit similar dissociation rates, complicating their discrimination. Overall, the estimates obtained after part 2 were of great quality with small errors. In absolute value, a difference of 1% was observed between the estimated and actual proportions at 12 °C. At 40 °C, the difference was of the order of 10%.

### Composition and concentration estimation on real sensorgram data

**Figure 9:**
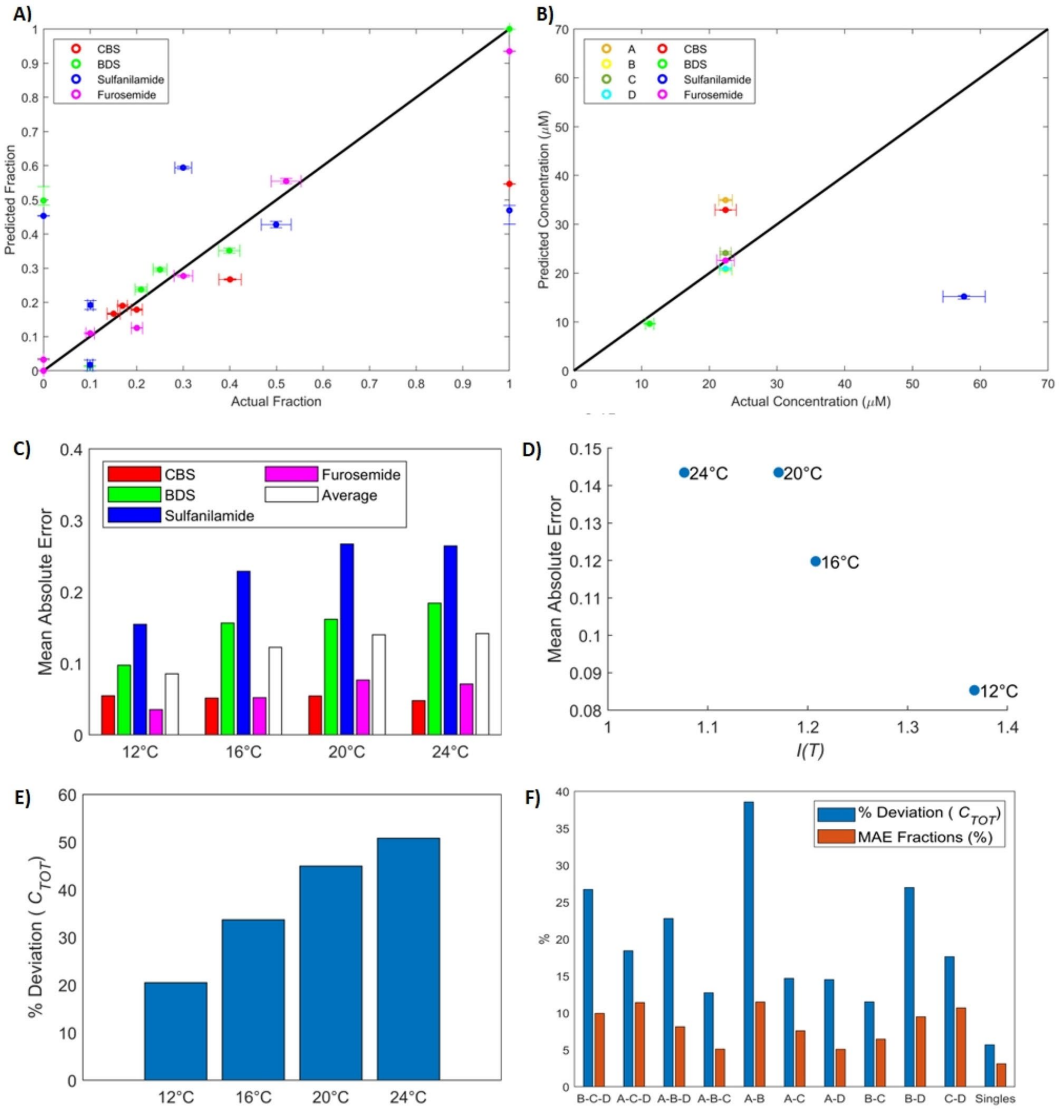
Performance of the composition and concentration estimation algorithm. (**A**) Calculated fractions with respect to actual fractions of the four analytes in each of the 8 mixtures detailed in Table 1. These fractions were estimated from sensorgrams at 12 °C only with kinetic parameters identified from data set (B–C–D). (B) Calculated concentration with respect to actual concentration for each of the 8 mixtures. These were estimated along the fractions using sensorgrams at 12 °C only with kinetic parameters identified from data set (B–C–D). (**C**) Mean absolute error of estimated fractions for each analyte at each temperature. The composition estimation algorithm was used independently for each set of kinetic parameters corresponding to each data set (all combinations of 2 and 3 mixtures) and each temperature, and mean absolute errors were averaged across all data sets and mixtures. (**D**) Mean absolute error (averaged across all data sets) with respect to the performance indicator. Annotations on the graph indicate the corresponding temperature. (**E**) Deviation (in %) between estimated concentrations and actual concentrations (averaged across all data sets) with respect to temperature. (**F**) Deviation of the concentration and mean absolute error of the fractions with respect to the data set that was used to identify the kinetics. Those were taken at 12 °C only. In this figure, ‘singles’ refers to single-analyte fits.

Next, we used the kinetic parameters sets identified with real sensorgrams (Tables S1 and S4 in Supplementary Materials) to estimate the composition and concentration of all eight mixtures described in Table 1. This was performed independently for every parameter set and every temperature. The algorithm detailed in the “Composition estimation” section was used. An example is shown in Fig. 9A,B. The kinetic parameters identified from data set B–C–D were used here, and only sensorgrams at 12 °C were fed to the algorithm. The vertical error bars in these plots correspond to asymmetric confidence intervals found with a method described in Supplementary Materials. The horizontal error bars were found by propagating the systematic error of the pipettes (Pipetman Neo P10, P20, P200 and P1000) and the balance (Dever Instrument Company AA-160) that were used to conduct the experiments. The small vertical error bars are mainly due to the large number of data points that are considered (injections of 7 concentrations, in duplicate with 10 points per second). For the most part, points are well aligned with the central line in these plots, indicating proper estimates. The experimental data corresponding to pure sulfanilamide were the most difficult to elucidate. This is mainly because the identified kinetic parameters of sulfanilamide exhibited the largest deviations from single-analyte estimates for this data set and temperature (~ 14% for the association rate and ~ 18% for the dissociation rate). The kinetic parameters of CBS, BDS and furosemide are relatively well identified (deviations between 0 and 11%). So are maximal responses (deviations below 10% for all four analytes). It appears that imprecisions in the identified kinetics of sulfanilamide are the driving force of the estimation errors. The fraction of sulfanilamide is overestimated in mixture A and pure CBS, leading to higher concentration estimates for these mixtures to compensate for sulfanilamide’s lower affinity for CAII. The other 5 mixtures were well identified. It might not be surprising that mixtures B, C and D were correctly elucidated, as those were used to identify the kinetics in this case study. BDS and furosemide have high affinities and their parameters were precisely identified, hence pure BDS and furosemide were also precisely estimated. BDS is falsely detected in the 100% sulfanilamide mixture, possibly because sulfanilamide and BDS have similar dissociation rates.

This corroborates Fig. 9C where sulfanilamide and BDS are shown to be the two analytes with the largest MAEs (averaged over all sets of kinetic parameters), no matter which injection temperature is considered. CBS and furosemide have much lower MAEs, with a weaker temperature dependence. As predicted by the simulations, we observed that performing the experiments at a lower temperature leads to more precise composition estimations. This cannot be explained by better kinetic parameters estimates at lower temperatures, because Figs. 5D and 7D showed no clear trend in parameter deviation with respect to temperature. Figure 9D shows that the MAE was also correlated to our suggested indicator. The same trend is observed for concentration estimates (Fig. 9E). This is easy to understand, as large errors in the fraction estimates can be somewhat compensated by the concentration estimation, and vice versa, as both the fractions and concentration are estimated concurrently.

Interestingly, the kinetic parameter sets identified from data sets A–C–D and C–D did not lead to larger estimation error. These data sets were highlighted during the parameter identification process, as they led to noticeably bigger deviations for the kinetic parameters of sulfanilamide. The other three analytes were well identified, however, and overall, these parameter sets can be used to estimate the composition and concentration with similar accuracy to other parameter sets. This is the case at 12 °C, as shown in Fig. 9F, and for other temperatures. The quality of the kinetic parameter estimates still plays an important part, as using the parameters identified from single-analyte experiments to estimate the composition and concentration led to the smallest errors (‘singles’ data set in Fig. 9F). The maximal response deviations from data set A–B were the largest. This led to large concentration estimation errors for mixtures aside from A and B. This data set led to the biggest deviations on the kinetics of furosemide, which was properly identified for all other data sets.

100% BDS and 100% sulfanilamide were the mixtures for which the estimation errors were the biggest on average. As previously mentioned, this is probably due to their similar dissociation rates, and sulfanilamide’s lower affinity for CAII.

Adding the concentration to the list of parameters to be identified adds a degree of freedom which can confound the estimation of the fractions. To evaluate this effect, we repeated the whole procedure, this time by constraining the value of the concentration to the correct experimental value. This ends up being practically the same as the algorithm proposed in^15^ for the case where concentration is known. The results are presented in detail in Supplementary Materials Figure S2. Fraction MAEs were approximately two-fold lower when the concentration is known (~ 4% vs ~ 8%). Removing a parameter to be fitted increases the precision of the optimization algorithm, resulting in smaller confidence intervals. Providing information to the algorithm facilitates convergence to the correct fractions as long as the set of kinetic parameters used is adequate. Larger MAEs were obtained with data set C–D when the concentrations are known, mainly driven by sulfanilamide whose parameters presented high deviations in this fit, which could not be compensated for by the concentration estimate.

Measuring the total concentration of all analytes prior to composition estimation (through an SPR experiment or otherwise), if possible, leads to more precise composition estimates. To do so via SPR, it is necessary that the analytes have the same refractive index increment. For proteins, this would mean similar molecular weights (for example different glycosylation or other post-translation-modifications of a same protein). One would first produce a calibration curve of the initial slope of a mass transport limited sensorgram with respect to the concentration of analyte using an analyte mixture of known concentration. The concentration of an unknown mixture could then be deduced from a mass transport limited sensorgram of the unknown mixture^19,34–36^.

### Analyte pooling for partial composition assessment

The previous sections showed promising results for the identification of kinetic parameters of $N$ analytes with $M<N$ mixtures and for the estimation of the composition and the concentration of an unknown mixture of these analytes. However, in both cases, sulfanilamide is highlighted as problematic due to its lower affinity to CAII (by approximately an order of magnitude compared to the other three analytes) that makes its contribution to the SPR response marginal unless it occupies a dominating proportion of the mixture. It also exhibits a similar dissociation rate as that of BDS, leading to confounding these two analytes when estimating mixture compositions.

When one or more analytes have weak affinities and/or similar kinetics leading to poorer fraction estimates, one solution is to construct analyte pools by regrouping two or more analytes into one. This can potentially lead to more relevant estimates for the pooled analyte, as the pool might have a more appreciable contribution than each of the individual pooled analytes. This might not affect the identification of other non-pooled analytes which already have high affinities and contributions to the SPR response.

**Figure 10:**
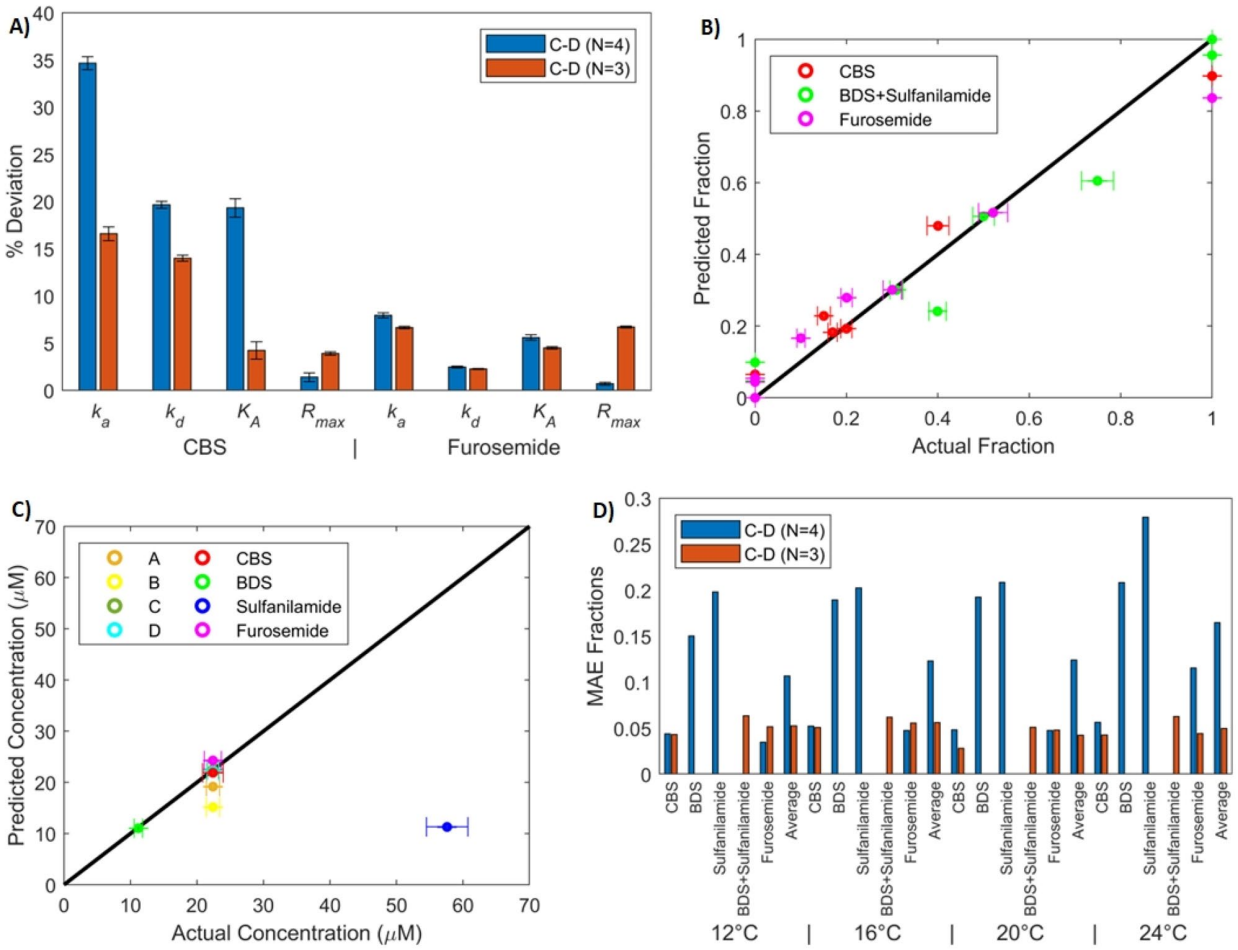
Performance of the kinetics identification and composition and concentration estimation algorithms when pooling BDS and sulfanilamide. (**A**) Deviation between the parameters (${k}_{a}$, ${k}_{d}$, ${K}_{A}$ and ${R}_{max}$) identified from single-analyte experiments and parameters identified by fitting the multi-analyte model at four temperatures with data set (C–D) for CBS and furosemide. For these fits, the total concentration and fractions of all analytes are assumed to be known (with ${F}_{pool}={F}_{BDS}+{F}_{sulfanilamide}$). Deviations were averaged over all temperatures. Error bars were computed by propagating the standard error of the parameters identified from single-analyte and multi-analyte experiments. (**B**) Calculated fractions with respect to actual fractions of the three analytes in each of the 8 mixtures. These fractions were estimated from sensorgrams at 12 °C only. (**C**) Calculated concentration with respect to actual concentration for each of the 8 mixtures. These were estimated along the fractions using sensorgrams at 12 °C only. (**D**) Mean absolute error of estimated fractions for each analyte at each temperature. The composition estimation algorithm was used independently with pooling of BDS and sulfanilamide and without pooling. Averages over all 8 mixtures are reported.

Applying this concept in our case study, we decided to pool sulfanilamide with BDS. This reduces the number of analytes to three. To remain within the scope of the parameter identification algorithm proposed in this study ($1<M<N$), we should have analyzed data sets containing two mixtures. We chose data set C–D, as both of these mixtures contained a small fraction of sulfanilamide, leading to big deviations in the identified kinetic parameters for this analyte. We used our parameter identification algorithm while considering ${F}_{pool}={F}_{BDS}+{F}_{sulfanilamide}$. Results are reported in Fig. 10A and in Supplementary Materials Table S6. The kinetics of CBS and furosemide were properly identified, and the kinetics of the pooled analyte closely matched those of BDS. This is not surprising, since the contribution of the pool to the SPR response is almost exclusively due to BDS. Interestingly, the deviations on the kinetics and affinity of CBS were lower with the pooled analyte than without it and they were similar for furosemide. Maximal responses exhibited bigger deviations when pooling, but they remained well below 10%. This suggests that pooling some analytes does not prevent proper identification of the non-pooled analytes. The χ^2^ with pooling was slightly higher than without (0.0249 vs 0.0241) as removing parameters necessarily leads to bigger residues. However, they exhibited no obvious trend and the model still properly depicted the data.

We then used this new set of identified kinetic parameters to estimate the composition of the 8 mixtures independently for each temperature. Example results are shown in Fig. 10B,C for an injection temperature of 12 °C.

The MAEs on fractions were lower when pooling. Of interest, the MAEs relative to CBS were slightly lower with pooling while those of furosemide were similar (see Fig. 10D). Deviations in the estimated concentrations were almost identical with and without pooling. The mixture with the highest concentration deviation was 100% sulfanilamide. This mixture is appropriately estimated to be composed of the pooled analyte almost exclusively (95% pool + 5% furosemide). However, since the pooled analyte’s affinity resembles that of BDS and not that of sulfanilamide, the estimated concentration is found to be almost five times lower than the real concentration. Without pooling, the mixture is estimated to be composed of (75% BDS and 25% sulfanilamide), also leading to a fivefold lower concentration estimate.

In summary, pooling a low affinity analyte and a strong affinity analyte with a similar dissociation rate made the identification of the other analytes equally or more precise while enabling better composition estimates. The kinetic parameter identification may not work as well if the data set used to fit the multi-analyte model is composed of mixtures in which the low affinity analyte has a dominating proportion. For example, repeating the procedure with data set A–B did not lead to appreciable gains in parameter or composition estimation performance. In these mixtures, sulfanilamide is present in high proportions (respectively 30% and 50%) and has an appreciable contribution to the SPR response.

## Discussion

This study pertains to the analysis of analyte mixtures by SPR biosensing. Those are ill-suited to traditional single-analyte studies, as analyte heterogeneity complexifies the recorded SPR signals and renders the use of a single Langmuir binding isotherm inadequate for data analysis. Using a kinetic model that properly describes the actual system is essential if one aims to uncover meaningful information on the interaction behavior. Moreover, the ability to analyze multi-analyte samples enables mixture composition estimation, which could prove a powerful tool to streamline bioprocess monitoring and development.

Carbonic anhydrase II (CAII) was used as the immobilized ligand in our experiments because of its well-known ability to form a simple 1:1 interaction with several small compounds with varying kinetic rates and affinities. Amongst CAII binders, we selected $N=4$ analytes: CBS, BDS, sulfanilamide and furosemide. To simulate a realistic situation, we constructed four mixtures of these analytes spanning a wide range of compositions (Table 1). The SPR response recorded when injecting each mixture on a CAII-immobilized surface should follow a multi-analyte 1:1 binding model, where every analyte competes to bind CAII. In this paper, we suggest two algorithms. The first one aims to identify the kinetic parameters of $N$ analytes with $M\le N$ mixtures of the analytes. To accomplish this, the mixtures must be injected at a minimum of $N/M$ temperatures, and the composition and concentration of the mixtures must be known (Fig. 1). The second algorithm seeks to estimate the composition and the concentration of a mixture, given that the kinetics have been identified previously (Fig. 2). If more than one concentration is injected, the dilution rate used to create the dilution series must be known.

We chose four injection temperatures: 12, 16, 20 and 24 °C as they allow full dissociation within a reasonable time while still exhibiting sufficient curvature during the association phase. We first injected pure samples of the four analytes at each temperature to obtain standard values of their actual kinetics. This allowed us to predict the contribution of each analyte in the four mixtures by solving the multi-analyte model ODEs (Fig. 3). We then injected the mixtures and utilized our first algorithm to identify the kinetics of the four analytes with three mixtures (Table S1). Fits to real sensorgram data were shown to be excellent (Fig. 4), and the identified kinetic parameters closely matched those obtained via single-analyte experiments, especially when sulfanilamide has an appreciable contribution to the SPR response (Fig. 5). Having identified the kinetics and affinities at each temperature, thermodynamic modeling became possible via the Eyring and Van’t Hoff equations (Fig. 6 and Table S2). The procedure was then repeated with combinations of only two mixtures (Fig. 7 and Table S4). The identified parameters still closely matched those from single-analyte experiments, but with slightly larger deviations than when using three mixtures to fit the model.

We then focused on our second algorithm. We showed, via simulations, that the algorithm performed better when the unknown mixture is injected at lower temperatures, or at a temperature for which the dissociation rates of the analytes are more disparate (Fig. 8). We then applied our second algorithm on real sensorgram data and obtained relatively precise estimates using 12 °C sensorgrams (Fig. 9). Throughout our study, sulfanilamide was highlighted as problematic because of its low affinity to CAII, leading to small contributions to the SPR response and a greater difficulty identifying its kinetics or estimating its fraction in an unknown mixture. It also has a very similar dissociation rate to that of BDS, potentially leading to confounding both analytes. We showed that considering them both as only one analyte (i.e., pooling them) could lead to a better performance of both of our algorithms, as long as the mixtures used to identify the kinetics contain little sulfanilamide (Fig. 10).

The merit of this current study compared to previous work on mixture analysis via SPR^15^ lies in the ability to reduce the number of mixtures required to extract the kinetics ($M\le N$ instead of $M=N$). When working with mixtures of compounds that are difficult to separate, it might be difficult to obtain multiple, different and linearly independent mixtures. On the other hand, injecting the available mixtures at several temperatures is a standard feature on most SPR biosensors. The only downside being longer SPR experiments due to stabilization periods required between temperature changes. Moreover, we showed that it is possible to estimate the concentration along the mixture composition, which was never reported before.

The multi-analyte kinetic model is structurally identifiable only locally rather than globally^32^. Briefly, this means that several (but a finite number of) sets of kinetic parameters may lead to the same predicted SPR response. To select a biologically meaningful solution, we proposed a preliminary subroutine (part 1 of our parameter identification algorithm) to provide adequate starting points for the optimization over the whole sensorgrams (part 2 of the algorithm). This is significant, since we have shown that a poor starting point may lead to convergence towards a solution that is further away from kinetic parameters derived from single-analyte experiments in the “Importance of the Initial Estimates for Parameter Identification” section and Supplementary Materials Table S3.

The structural identifiability of part 1 of the parameter identification algorithm was briefly discussed in "Required data" section. Because summation (here of exponential decays) is commutative, at least two mixtures are necessary so that the $M$ by $N$ matrix of ${Z}_{i,m}$ and $N$ by $1$ vector of ${k}_{d,i}$ describing the dissociation phase of all mixtures are ordered the same way (in terms of the analytes) as the $M$ by $N$ matrix of analyte fractions ${F}_{i,m}$. The latter is assumed to be known and is fed to the algorithm. Additionally, no analyte couple should have perfectly correlated fractions in the available mixtures. If this condition is not met, the product of ${K}_{A,i}$ and ${R}_{max,i}$ could be adjusted so that different orders could lead to the same set of ${Z}_{i}$, obfuscating the identification of ${K}_{A,i}$ and ${R}_{max,i}$.

Another important concept to consider is practical identifiability. A non-identifiable model that can be made identifiable by adding data or reducing measurement noise is deemed practically non-identifiable. While there is no universal method to test practical identifiability, an elegant one consists in confirming that no fitted parameter has infinite confidence intervals. This can be done, for example, by disturbing the optimal solution one parameter at a time and re-optimizing, which leads to a profile likelihood^37,38^. We used the same method we used previously to derive confidence intervals on the parameters derived from part 1 of the algorithm (as detailed in Supplementary Materials), and found finite confidence intervals for all parameters, be it for data sets consisting of three or two mixtures (Fig. S3 in Supplementary Materials). Hence, the model of part 1 of the algorithm is deemed practically identifiable with the available amount of data and the noise level of our SPR instrument.

The goal of this study was to establish a proof of concept for both algorithms, not to optimize their use. Indeed, the parameter identification algorithm could theoretically work with only two injected concentrations (just enough to identify the observed affinity and maximal response) while seven were injected. The duration of the association and dissociation phases could also be shortened, as long as an equilibrium plateau is still reached and the response goes back to zero at the end of the sensorgram, so as to avoid the need for a regeneration step (in our experimental system). Some previous studies have aimed to optimize these parameters online for single-analyte experiments^9,11^. Furthermore, in our case study where $N=4$ and $M=3$ (or $M=2$), only two temperatures were theoretically necessary (${N}_{T}\ge N/M$). This is dictated by the system of linear equations in (19), which needs to be solved to obtain a first estimate of the maximal responses. We recommend using more temperatures than the minimum required to help counter the effects of experimental error and measurement noise, and thus obtain an accurate solution of Eq. (19). Moreover, the selected injection temperatures should be spaced enough so that the temperature-driven differences in binding behavior are more pronounced than the experimental error and noise, again to facilitate solving Eq. (19). The same reasoning can be applied to the number of mixtures, with more mixtures leading to more accurate parameter estimates, as we observed in this study.

Low temperatures slow the kinetics, which may prevent reaching an equilibrium state in a reasonable injection time, whereas high temperatures may result in ‘rectangular’ sensorgrams which contain little information in their curvature. Hence, extreme temperatures should be avoided. The selection of temperatures to perform parameter identification will be dependent on the studied binders but beginning towards the middle of the temperature range allowable by the SPR biosensor and making positive and negative steps of 4 or 5 degrees may be a good general starting point.

The experimental error was estimated by performing the same dilution series of the same analyte three times, injecting them on the SPR surface, and identifying the resulting kinetic parameters. Deviations of up to 10% were obtained between such triplicates. Our parameter identification algorithm led to deviations of this scale when using three mixtures, whereas only using two mixtures led to deviations that are twice as high. Such seems to be the cost of reducing the amount of available data.

Our model considers a specific maximal response (${R}_{max,i}$) for all analytes. This was necessary for our system, as small molecular weight compounds, such as those used as analytes in this study, exhibit analyte-specific refractive index increments^12^. For high molecular weight compounds, such as proteins, ${R}_{max,i}$ should be linearly proportional to the molecular weight^39–41^. This would simplify the parameter identification algorithm, as only one ${R}_{max}$ would need to be included, provided that molecular weights are known. For proteins, a very small temperature dependence of the refractive index increment has been reported^42–44^. However, it has been modelled using the same temperature-dependent function for multiple proteins between 10 and 25 °C^42,43^. Hence, the standardization procedure detailed in section "Furosemide standard injections" should allow the temperature invariant ${R}_{max,i}$ assumption.

Using our algorithm could save experimental time when performing thermodynamic analyses. Indeed, rather than injecting all $N$ analytes at multiple temperatures, one could inject $M<N$ mixtures instead. In both cases, kinetic parameters and affinities could be identified at all temperatures, enabling the construction of Eyring and Van’t Hoff plots from which entropic and enthalpic changes could be extracted. Incidentally, the Eyring and Van’t Hoff plots obtained with single-analyte experiments showed high ${R}^{2}$ ($>99\%$), indicating that the hypothesis of temperature independent entropic and enthalpic changes is appropriate for our system.

For the unknown mixture composition and concentration determination, sensorgrams obtained at only one temperature were used. Using sensorgrams from more than one temperature might lead to more accurate estimates, but it seems ill-fitting to the goal of this algorithm, which is to rapidly determine the composition. Indeed, changing the injection temperature requires the biosensor to restabilize, which takes at least one hour for a precise and stable temperature. It seems more realistic to envision the case where a user would inject multiple unknown samples subsequently at the same temperature. For the parameter identification, one must perform experiments at multiple temperatures, meaning restabilizing the instrument multiple times. This process could prove lengthy, but once the parameters have been identified, they can be used to analyze a large number of unknown mixtures more promptly afterwards.

Identifying specific binding kinetics may be daunting when characterizing macromolecules that are difficult to purify. For instance, it is well documented that immunoglobulin G (IgG) production in bioreactor leads to a heterogeneous distribution of IgG glycoforms^25^. The identity of the glycan groups added to the IgG Fc region greatly influences therapeutic efficacy, mainly by affecting binding to Fcγ receptors present on effector immune cells^20–26^. The IgG-FcγR interaction can be measured by SPR biosensing^27^. The abundance of each glycoform can be deduced from mass spectrometry analysis, for example^20–26^. Hence, an algorithm similar to that proposed in^15^ could be used to uncover glycoform-specific kinetics. However, the number of different glycoforms is usually quite large, and while different mixtures of glycoforms can be created via bioengineering methods^25^, by varying cell culture conditions^45^ and by using different cell lines^46^, reducing the number of mixtures needed remains necessary. Using our approach, this could be achieved by injecting the available mixtures at different temperatures, or by pooling glycoforms that have a lower affinity for the FcγR. Having uncovered the kinetics, our second algorithm could be used to estimate the glycosylation profile and concentration of a subsequent sample. We believe this could pave a new way for SPR to be used in product quality monitoring.

Our analysis framework could be applied to study heterogeneity in the analyte solution hailing from factors other than glycosylation, such as differences in size or folding, for example.

We propose an algorithm that can estimate the concentration, as long as the dilution factors used to create the dilution series are known (if more than one concentration was injected). However, knowing the concentration still leads to more precise composition estimates, as errors in the composition estimate can be compensated for by errors in the concentration estimate, and vice versa. The active concentration can be found by using a surface with a high density of immobilized ligand and a low flow rate, so that the binding process is limited by mass transport limitations^36,47–49^.

The system is assumed to be limited by the binding kinetics in both of our algorithms, rather than mass transport. If mass transport limitations are only moderate, the system should behave similarly to a kinetically limited system, except with slower kinetics. There should not be an effect on the binding affinity nor the equilibrium behavior. The algorithms presented here might be usable on systems moderately limited by mass transport limitations (as long as equilibrium may be reached), but the kinetics uncovered would be biased^11,48,50^.

## Conclusion

We extended the previously existing framework for the characterization of analyte mixtures via SPR biosensing. This was achieved by taking advantage of the ability of SPR biosensors to perform experiments at different temperatures. The developed algorithms extracted analyte-specific kinetics from mixtures of analytes and then used these kinetic parameters to identify the composition of other unknown mixtures. The algorithms were found to be precise and robust. Performing experiments at multiple temperatures effectively reduced the number of mixtures that was required to extract analyte-specific kinetics. A couple limitations remain in our analysis framework. First, a simple 1:1 binding model is assumed, which might not be appropriate for all systems. Second, the identification of the kinetics still requires prior knowledge of the composition and the concentration of the mixtures used in our first algorithm, which may be obtained with orthogonal characterization techniques.

We believe that mixture analysis opens a new avenue for the use of SPR both as a valuable tool to facilitate and accelerate the development of bioprocesses and as a monitoring tool for quality control. With more emphasis being put on process analytical tools in recent years^16,19^, developing new data analysis methods and experimental assays will ensure SPR biosensing remain an asset for research and industry.

## Supplementary Information

Supplementary Information.

## Data Availability

The datasets used and/or analysed during the current study available from the corresponding author on reasonable request.
