# Supplementary material for: Multi-temperature experiments to ease analysis of heterogeneous binder solutions by surface plasmon resonance biosensing
Source: Sci Rep. 2022 Aug 24;12:14401. doi: 10.1038/s41598-022-18450-y (PMC9402583; doi:10.1038/s41598-022-18450-y)
Supplement: Supplementary file 1 — Supplementary Information. [file 41598_2022_18450_MOESM1_ESM.pdf]

Title: Multi-Temperature Experiments to Ease Analysis of Heterogeneous Binder Solutions by Surface Plasmon Resonance Biosensing

Authors

Jimmy Gaudreault[1], Yves Durocher[2], Olivier Henry[1,*], Gregory De Crescenzo[1,*]

*: These authors contributed equally to this work.

[1] Department of Chemical Engineering, Polytechnique Montréal. P.O. Box 6079, Centre-ville Station, H3C 3A7 Montréal, Québec, Canada.

[2] Life Sciences | NRC Human Health Therapeutics Portfolio, Building Montreal-Royalmount, National Research Council Canada, Montreal (QC), Canada H4P 2R2.

Correspondence and requests for materials should be addressed to O. H. (email: olivier.henry@polymtl.ca) or G.D.C. (email: gregory.decrescenzo@polymtl.ca)

Author Contributions

J.G. performed all experiments and data analysis, Y.D. provided mentorship, O.H. and G.D.C contributed equally to the supervision of this work. All authors reviewed the manuscript.

Availability of Data and Materials

The datasets used and/or analysed during the current study available from the corresponding author on reasonable request.

Supplementary Materials

1. First Part of the Parameter Identification Algorithm

Boundaries on the Identified Parameters

We impose the following boundaries on the parameters to be identified:

1. Equilibrium contributions must be between 0 and 1:

$$0 \leq Z_{i,m,T} \leq 1 \quad \forall\, m, T$$

2. The dissociation rates are positive:

$$k_{d,i,T_1} > 0 \quad \forall\, i$$

3. The dissociation rates increase with temperature. From the Eyring equation), it follows that:

$$C_{4,i} < 0 \quad \forall\, i$$

Constraints on the Identified Parameters

We consider three types of constraints on the identified parameters:

1. The sum of the equilibrium contributions is always 1 for all mixtures and temperatures ($M \cdot N_T$ linear equality constraints):

$$\sum_{i=1}^{N} Z_{i,m,T} = 1 \quad \forall\, m, T$$

In terms of the adjusted parameters $Z_{i,m_1,T}$, we have:

$$\sum_{i=1}^{N} Z_{i,m_1,T} \frac{F_{i,m}}{F_{i,m_1}} \frac{\left(R_{max,obs} K_{A,obs}\right)_{m_1,T}}{\left(R_{max,obs} K_{A,obs}\right)_{m,T}} = 1 \quad \forall\, m, T$$

2. The association rates $k_{a,i}$ increase with temperature. The expression of this constraint with respect to the identified parameters is described lower. This leads to $N \cdot (N_T - 1)$ nonlinear inequality constraints.

3. The affinity $K_{A,i}$ varies monotonously with respect to temperature (either increases or decreases, depending on if the dissociation or association rate temperature dependence is greater). The expression of this constraint according to the identified parameters is described lower. This leads to $N \cdot (N_T - 2)$ nonlinear inequality constraints.

Constraints on the Association Rates

The association rates $k_{a,i}$ increase with temperature. One can express $k_{a,i,T}$ via the Eyring equation-derived definition of the dissociation rates with a reference temperature T_1:

$$k_{a,i,T} = K_{A,i,T} k_{d,i,T_1} \frac{T}{T_1} \exp\left(C_{4,i}\left(\frac{1}{T} - \frac{1}{T_1}\right)\right)$$

We take two temperatures T_A and T_B. If $T_B > T_A$, it follows that $k_{a,i,T_B} > k_{a,i,T_A}$ $\forall i$:

$$K_{A,i,T_B} k_{d,i,T_1} \frac{T_B}{T_1} \exp\left(C_{4,i}\left(\frac{1}{T_B} - \frac{1}{T_1}\right)\right) > K_{A,i,T_A} k_{d,i,T_1} \frac{T_A}{T_1} \exp\left(C_{4,i}\left(\frac{1}{T_A} - \frac{1}{T_1}\right)\right)$$

$$K_{A,i,T_B} T_B \exp\left(C_{4,i}\left(\frac{1}{T_B} - \frac{1}{T_1}\right)\right) > K_{A,i,T_A} T_A \exp\left(C_{4,i}\left(\frac{1}{T_A} - \frac{1}{T_1}\right)\right)$$

We know that the affinity can be obtained from the equilibrium contribution:

$$K_{A,i,T} = \frac{Z_{i,m_1,T}\left(K_{A,obs} R_{max,obs}\right)_{m_1,T}}{F_{i,m_1} R_{max,i}} \quad \forall i, T$$

By inputting this definition in the inequality, we obtain:

$$\frac{Z_{i,m_1,T_B}\left(K_{A,obs} R_{max,obs}\right)_{m_1,T_B} T_B \exp\left(C_{4,i}\left(\frac{1}{T_B} - \frac{1}{T_1}\right)\right)}{F_{i,m_1} R_{max,i}} > \frac{Z_{i,m_1,T_A}\left(K_{A,obs} R_{max,obs}\right)_{m_1,T_A} T_A \exp\left(C_{4,i}\left(\frac{1}{T_A} - \frac{1}{T_1}\right)\right)}{F_{i,m_1} R_{max,i}}$$

$$Z_{i,m_1,T_B}\left(K_{A,obs} R_{max,obs}\right)_{m_1,T_B} T_B \exp\left(\frac{C_{4,i}}{T_B}\right) > Z_{i,m_1,T_A}\left(K_{A,obs} R_{max,obs}\right)_{m_1,T_A} T_A \exp\left(\frac{C_{4,i}}{T_A}\right)$$

$$Z_{i,m_1,T_B} \frac{\left(K_{A,obs} R_{max,obs}\right)_{m_1,T_B}}{\left(K_{A,obs} R_{max,obs}\right)_{m_1,T_A}} \frac{T_B}{T_A} \exp\left(C_{4,i}\left(\frac{1}{T_B} - \frac{1}{T_A}\right)\right) - Z_{i,m_1,T_A} > 0$$

This leads to $N \cdot (N_T - 1)$ nonlinear inequality constraints.

Constraints on the Affinities

The affinities $K_{A,i}$ vary monotonously with respect to temperature. We can express the affinity via the equilibrium contribution:

$$K_{A,i,T} = \frac{Z_{i,m_1,T}\left(K_{A,obs} R_{max,obs}\right)_{m_1,T}}{F_{i,m_1} R_{max,i}} \quad \forall i, T$$

As F_{i,M_1} and $R_{max,i}$ are not temperature dependent, one only has to verify that the numerator $G_{i,T} = Z_{i,m_1,T}\left(K_{A,obs}R_{max,obs}\right)_{m_1,T}$ only increases or decreases with temperature. We take three temperatures $T_C > T_B > T_A$ to obtain the following constraint:

$$\left(G_{i,T_C} - G_{i,T_B}\right)\left(G_{i,T_B} - G_{i,T_A}\right) > 0 \quad \forall i, T_A, T_B, T_C$$

This gives a total of $N \cdot (N_T - 2)$ nonlinear inequality constraints.

Starting Point of the Optimization Routine

The order of magnitude of the $Z_{i,m,T}$ is known as they are required to be comprised between 0 and 1 and sum to one. Hence, an obvious starting would be $1/N$ for all analytes. An appropriate starting point for k_{d,i,T_1} and $C_{4,i}$ is not as obvious, a priori. SPR biosensors can measure dissociation rates ranging from approximately 10^{-4} to 0.5 s^{-1}, and the $C_{4,i}$ are a function of the enthalpy of the dissociation pseudo-reaction. Given that the dissociation phase is long enough to reach a null response for all sensorgrams, we suggest a starting point based on the data and a coarse assumption. This will give in turn a coarse starting point, but of a reasonable order of magnitude.

We take the integral of the dissociation phase (ranging from the beginning of the dissociation phase t_{diss} to the end of the sensorgram t_{end}) of the normalized SPR sensorgrams ($I_{m,T}$):

$$I_{m,T} = \int_{t_{diss}}^{t_{end}} R_{norm,m,T}\,(t)\,dt = \int_{t_{start}}^{t_{end}} \sum_{i=1}^{N} Z_{i,m} \exp\left(-k_{d,i,T}\ t\right) dt$$

If t_{end} is such that the SPR response is approximately zero towards the end of the sensorgram, we obtain:

$$I_{m,T} = \sum_{i=1}^{N} \frac{Z_{i,m}}{k_{d,i,T}}$$

We express the previous equation with respect to a reference temperature T_1 to obtain:

$$I_{m,T} = \sum_{i=1}^{N} \frac{Z_{i,m}}{k_{d,i,T_1}} \frac{T_1}{T} \exp\left(C_{4,i}\left(\frac{1}{T_1} - \frac{1}{T}\right)\right) \quad \forall\, m, T$$

We consider the case where all analytes share the same $k_{d,i,T_1} = K$ and $C_{4,i} = C$. We obtain:

$$I_{m,T} = \frac{T_1}{KT} \exp(C\tau) \quad \forall\, m, T$$

With $\tau = \left(\frac{1}{T_1} - \frac{1}{T}\right)$. From there, we linearize to obtain:

$$\ln\left(\frac{I_{m,T}T}{T_1}\right) = -\ln(K) + C\tau$$

Regression of $\ln\left(\frac{I_{m,T}T}{T_1}\right)$ with respect to τ by considering all sensorgrams (all mixtures and temperatures) leads to estimates of K and C which are themselves coarse estimates of the k_{d,i,T_1} and the $C_{4,i}$, respectively. This procedure leads to starting values of the right order of magnitude for the suggested optimization routine.

2. Confidence Intervals on the Kinetic Parameters

The confidence intervals can be computed based on the standard error [7,9,15]. For the kth parameter, the standard error is given by:

$$SE(k) = \sqrt{[H^{-1}]_{k,k}\,\chi^2}$$

Where $[H^{-1}]_{k,k}$ is the kth element on the diagonal of the inverse of the Hessian matrix. χ^2 is a function of the number of identified parameters $p = N \cdot (2N_T + 1)$ and of the number of data points n:

$$\chi^2 = \frac{\sum_{s=1}^{S}\sum_{t=1}^{T}\left(R_{TOT,meas}^{s,t} - R_{TOT,pred}^{s,t}\right)^2}{n-p}$$

The computation of the Hessian matrix is detailed in the Supplementary Materials of [15] for the case of a single injection temperature. The extension to multi-temperature experiments is given lower. Once the standard error of a given parameter k has been obtained, the $100(1-\alpha)\%$ confidence interval for that parameter is computed via Student's T distribution:

$$k = k_{pred} \pm t_{n-p,\alpha/2} \cdot SE(k)$$

Hessian Matrix with N analytes and N_T Injection Temperatures

The Hessian matrix can be approximated by the following sum on all time steps t of all available sensorgrams s (sensorgrams corresponding to different mixtures and temperatures):

$$H \approx \sum_{s}\sum_{t}\left(\frac{\partial R_{TOT,pred}^{s,t}}{\partial\theta}\right)^{T}\left(\frac{\partial R_{TOT,pred}^{s,t}}{\partial\theta}\right)$$

Where the parameter vector θ is itself a combination of vectors:

$$\theta = [\boldsymbol{k_a'}, \boldsymbol{k_d'}\, \boldsymbol{R_{max}'}\,]'$$

$$\boldsymbol{k_a} = \left[k_{a,1,1}, \dots, k_{a,N,1}, k_{a,1,2}, \dots, k_{a,N,2}, \dots, k_{a,1,N_T}, \dots, k_{a,N,N_T}\right]'$$

$$\boldsymbol{k_d} = \left[k_{d,1,1}, \dots, k_{d,N,1}, k_{d,1,2}, \dots, k_{d,N,2}, \dots, k_{d,1,N_T}, \dots, k_{d,N,N_T}\right]'$$

$$\boldsymbol{R_{max}} = \left[R_{max,1}, \dots, R_{max,N}\right]'$$

The gradient on the total predicted response corresponds to the sum of the gradient on the response of each analyte:

$$\frac{\partial R_{TOT,pred}}{\partial\theta} = \sum_{i=1}^{N}\frac{\partial R_{pred,i}}{\partial\theta}$$

The derivatives above can be evaluated by solving the following ODEs along with the system of ODE in :

$$\frac{\mathrm{d}R}{\mathrm{d}t} = f(R,\theta)$$

$$\frac{\mathrm{d}}{\mathrm{d}t}\frac{\mathrm{d}R}{\mathrm{d}\theta} = \frac{\partial f}{\partial \theta} + \frac{\partial f}{\partial R}\frac{\partial R}{\partial \theta}$$

$$R(0) = [R_1(0), \dots R_N(0)]' = [0,\cdots,0]'$$

$$\left.\frac{\partial R}{\partial \theta}\right|_{t=0} = 0$$

$$f_i = k_{ai}F_iC_{TOT}R_{max,i}\left(1 - \sum_{j=1}^{N}\frac{R_j}{R_{max,j}}\right) - k_{di}R_i \quad \forall\, i = 1,\dots,N$$

Here we present a way to compute the necessary gradient $\frac{\partial R_{TOT,pred}}{\partial \theta}$ to compute the Hessian matrix related to the estimation of the kinetic parameters. For a sensorgram at injection temperature T, we consider these 2 matrices with $\boldsymbol{R} = [R_1, \cdots R_N]'$:

$$\boldsymbol{X_{1,T}} = \begin{bmatrix} \frac{\partial R_1}{\partial k_{a,1,T}} & \cdots & \frac{\partial R_1}{\partial k_{a,N,T}} \\ \vdots & \ddots & \vdots \\ \frac{\partial R_N}{\partial k_{a,1,T}} & \cdots & \frac{\partial R_N}{\partial k_{a,N,T}} \end{bmatrix} = \frac{\partial \boldsymbol{R}}{\partial \boldsymbol{k_{a,T}}}$$

$$\boldsymbol{X_{2,T}} = \begin{bmatrix} \frac{\partial R_1}{\partial k_{d,1,T}} & \cdots & \frac{\partial R_1}{\partial k_{d,N,T}} \\ \vdots & \ddots & \vdots \\ \frac{\partial R_N}{\partial k_{d,1,T}} & \cdots & \frac{\partial R_N}{\partial k_{d,N,T}} \end{bmatrix} = \frac{\partial \boldsymbol{R}}{\partial \boldsymbol{k_{d,T}}}$$

Where $\boldsymbol{k_{a,T}}$ and $\boldsymbol{k_{d,T}}$ are the sections of vectors $\boldsymbol{k_a}$ and $\boldsymbol{k_d}$ that correspond to temperature T. We consider only those sections because kinetic rates at different temperatures have no effect on the predicted response at temperature T and hence corresponding derivatives will be zero. Maximal responses, however, are not temperature dependent. We consider a third matrix:

$$\boldsymbol{X_3} = \begin{bmatrix} \frac{\partial R_1}{\partial R_{max,1}} & \cdots & \frac{\partial R_1}{\partial R_{max,N}} \\ \vdots & \ddots & \vdots \\ \frac{\partial R_N}{\partial R_{max,1}} & \cdots & \frac{\partial R_N}{\partial R_{max,N}} \end{bmatrix} = \frac{\partial \boldsymbol{R}}{\partial \boldsymbol{R_{max}}}$$

Sensitivity with respect to $\boldsymbol{k_{a,i,T}}$:

$$\frac{\mathrm{d}\boldsymbol{X_{1,T}}}{\mathrm{d}t}(i,i) = \frac{\partial f_i}{\partial k_{a,i,T}} + \frac{\partial f_i}{\partial R_i}\cdot\frac{\partial R_i}{\partial k_{a,i,T}} + \sum_{j\neq i}^{N}\frac{\partial f_i}{\partial R_j}\cdot\frac{\partial R_j}{\partial k_{a,i,T}}$$

$$= \frac{\partial f_i}{\partial k_{a,i,T}} + \frac{\partial f_i}{\partial R_i} \cdot X_{1,T}(i,i) + \sum_{j \neq i}^{N} \frac{\partial f_i}{\partial R_j} \cdot X_{1,T}(j,i)$$

$$\frac{\mathrm{d}\boldsymbol{X}_{\boldsymbol{1},\boldsymbol{T}}}{\mathrm{d}t}(i,j) = \frac{\partial f_i}{\partial k_{a,j,T}} + \frac{\partial f_i}{\partial R_i} \cdot \frac{\partial R_i}{\partial k_{a,j,T}} + \sum_{k \neq i}^{N} \frac{\partial f_i}{\partial R_k} \cdot \frac{\partial R_k}{\partial k_{a,j,T}}$$

$$= \frac{\partial f_i}{\partial k_{a,j,T}} + \frac{\partial f_i}{\partial R_i} \cdot X_{1,T}(i,j) + \sum_{k \neq i}^{N} \frac{\partial f_i}{\partial R_k} \cdot X_{1,T}(k,j)$$

With partial derivatives (with $C_{TOT} = 0$ during the dissociation phase):

$$\frac{\partial f_i}{\partial R_i} = -k_{a,i,T} F_i C_{TOT} - k_{d,i,T}$$

$$\frac{\partial f_i}{\partial R_j} = -k_{a,i,T} F_i C_{TOT} \frac{R_{max,i}}{R_{max,j}}$$

$$\frac{\partial f_i}{\partial k_{a,i,T}} = F_i C_{TOT} R_{max,i} \left(1 - \sum_{j=1}^{N} \frac{R_j}{R_{max,j}} \right)$$

$$\frac{\partial f_i}{\partial k_{a,j,T}} = 0$$

We obtain:

$$\frac{\partial R_{TOT,pred}}{\partial \boldsymbol{k}_{\boldsymbol{a},\boldsymbol{T}}} = \begin{bmatrix} \sum_{i=1}^{N} \frac{\partial R_i}{\partial k_{a,1,T}} \\ \vdots \\ \sum_{i=1}^{N} \frac{\partial R_i}{\partial k_{a,N,T}} \end{bmatrix} = \begin{bmatrix} \frac{\partial R_{TOT,pred}}{\partial k_{a,1,T}} \\ \vdots \\ \frac{\partial R_{TOT,pred}}{\partial k_{a,N,T}} \end{bmatrix}$$

Sensitivity with respect to $\boldsymbol{k_{d,i}}$:

$$\frac{\mathrm{d}\boldsymbol{X}_{\boldsymbol{2},\boldsymbol{T}}}{\mathrm{d}t}(i,i) = \frac{\partial f_i}{\partial k_{d,i,T}} + \frac{\partial f_i}{\partial R_i} \cdot \frac{\partial R_i}{\partial k_{d,i,T}} + \sum_{j \neq i}^{N} \frac{\partial f_i}{\partial R_j} \cdot \frac{\partial R_j}{\partial k_{d,i,T}}$$

$$= \frac{\partial f_i}{\partial k_{d,i,T}} + \frac{\partial f_i}{\partial R_i} \cdot X_{2,T}(i,i) + \sum_{j \neq i}^{N} \frac{\partial f_i}{\partial R_j} \cdot X_{2,T}(j,i)$$

$$\frac{\mathrm{d}\boldsymbol{X}_{\mathbf{2},\boldsymbol{T}}}{\mathrm{d}t}(i,j) = \frac{\partial f_i}{\partial k_{d,j,T}} + \frac{\partial f_i}{\partial R_i} \cdot \frac{\partial R_i}{\partial k_{d,j,T}} + \sum_{k \neq i}^{N} \frac{\partial f_i}{\partial R_k} \cdot \frac{\partial R_k}{\partial k_{d,j,T}}$$

$$= \frac{\partial f_i}{\partial k_{d,j,T}} + \frac{\partial f_i}{\partial R_i} \cdot X_{2,T}(i,j) + \sum_{k \neq i}^{N} \frac{\partial f_i}{\partial R_k} \cdot X_{2,T}(k,j)$$

With partial derivatives:

$$\frac{\partial f_i}{\partial k_{d,i,T}} = -R_i$$

$$\frac{\partial f_i}{\partial k_{d,j,T}} = 0$$

We obtain:

$$\frac{\partial R_{TOT,pred}}{\partial \boldsymbol{k}_{\boldsymbol{d},\boldsymbol{T}}} = \begin{bmatrix} \sum_{i=1}^{N} \frac{\partial R_i}{\partial k_{d,1,T}} \\ \vdots \\ \sum_{i=1}^{N} \frac{\partial R_i}{\partial k_{d,N,T}} \end{bmatrix} = \begin{bmatrix} \frac{\partial R_{TOT,pred}}{\partial k_{d,1,T}} \\ \vdots \\ \frac{\partial R_{TOT,pred}}{\partial k_{d,N,T}} \end{bmatrix}$$

Sensitivity with respect to $\boldsymbol{R_{max,i}}$:

$$\frac{\mathrm{d}\boldsymbol{X}_{\mathbf{3}}}{\mathrm{d}t}(i,i) = \frac{\partial f_i}{\partial R_{max,i}} + \frac{\partial f_i}{\partial R_i} \cdot \frac{\partial R_i}{\partial R_{max,i}} + \sum_{j \neq i}^{N} \frac{\partial f_i}{\partial R_j} \cdot \frac{\partial R_j}{\partial R_{max,i}}$$

$$= \frac{\partial f_i}{\partial R_{max,i}} + \frac{\partial f_i}{\partial R_i} \cdot X_3(i,i) + \sum_{j \neq i}^{N} \frac{\partial f_i}{\partial R_j} \cdot X_3(j,i)$$

$$\frac{\mathrm{d}\boldsymbol{X}_{\mathbf{3}}}{\mathrm{d}t}(i,j) = \frac{\partial f_i}{\partial R_{max,j}} + \frac{\partial f_i}{\partial R_i} \cdot \frac{\partial R_i}{\partial R_{max,j}} + \sum_{k \neq i}^{N} \frac{\partial f_i}{\partial R_k} \cdot \frac{\partial R_k}{\partial R_{max,j}}$$

$$= \frac{\partial f_i}{\partial R_{max,j}} + \frac{\partial f_i}{\partial R_i} \cdot X_3(i,j) + \sum_{k \neq i}^{N} \frac{\partial f_i}{\partial R_k} \cdot X_3(k,j)$$

With partial derivatives:

$$\frac{\partial f_i}{\partial R_{max,i}} = k_{a,i,T} F_i C_{TOT} \left(1 - \sum_{j \neq i}^{N} \frac{R_j}{R_{max,j}}\right)$$

$$\frac{\partial f_i}{\partial R_{max,j}} = \frac{k_{a,i,T} F_i C_{TOT} R_{max,i}}{R_{max,j}^2} R_j$$

We obtain:

$$\frac{\partial R_{PRED,TOT}}{\partial R_{max}} = \begin{bmatrix} \sum_{i=1}^{N} \frac{\partial R_i}{\partial R_{max,1}} \\ \vdots \\ \sum_{i=1}^{N} \frac{\partial R_i}{\partial R_{max,N}} \end{bmatrix} = \begin{bmatrix} \frac{\partial R_{TOT,pred}}{\partial R_{max,1}} \\ \vdots \\ \frac{\partial R_{TOT,pred}}{\partial R_{max,N}} \end{bmatrix}$$

For each time point of each sensorgram, we obtain the gradient $\frac{\partial R_{TOT,pred}}{\partial \boldsymbol{\theta}}$. For injection temperature T, it contains the subsections computed using $\boldsymbol{X_{1,T}}$, $\boldsymbol{X_{2,T}}$ and $\boldsymbol{X_3}$ and zeros for every element corresponding to kinetic rates at other temperatures. Computing the gradient at every time step of every sensorgram in the data set (multiple mixtures and temperatures) is necessary to compute the Hessian matrix.

If bulk effect parameters (R_I, one per sensorgram) were added to the fitted model, additional elements must be added to $\frac{\partial R_{TOT,pred}}{\partial \boldsymbol{\theta}}$ as the parameter vector then contains as many additional elements as there are sensorgrams in the data set. For a given sensorgram s, the associated bulk effect parameter is noted $R_{I,s}$, and $\frac{\partial R_{TOT,pred}^{s}}{\partial \boldsymbol{R_{I,s}}}$ is equal to 1 for time steps during the association phase and 0 for time steps during the dissociation phase.

3. Confidence Intervals on the Fractions and Concentrations

A method to evaluate the confidence interval on the estimated fractions is given in [15] for the case of known total concentration. It leads to asymmetrical intervals so that the constraints on the F_i can be taken into account (contained between 0 and 1). We may use the same general method here, while considering $C_{TOT,REF}$ as an additional identified parameter. The method is based on the Fisher F statistic [15,51]. Taking into consideration the objective function in (26), we have:

$$\frac{J(\theta)|_{\theta_i=\theta_{i0}} - J(\hat{\theta})}{J(\hat{\theta})/(n-p)} \sim F(1, n-p)$$

Where $J(\hat{\theta})$ is the value of the objective function corresponding to the optimum point (estimated fractions and concentration) and $J(\theta)|_{\theta_i=\theta_{i0}}$ is the value of the objective function obtained by optimizing with an added constraint $\theta_i = \theta_{i0}$. The boundaries of the $100(1-\alpha)\%$ confidence interval are such that:

$$J(\theta)|_{\theta_i=\theta_{i0}} - J(\hat{\theta}) = F_{1-\alpha}(1, n-p) \cdot \frac{J(\hat{\theta})}{n-p}$$

$F_{1-\alpha}(1, n-p)$ is a quantile of the Fisher law with degrees of freedom in the parentheses. To find the upper boundary of the confidence interval of parameter i, we restart the optimization by disturbing parameter i such that $\theta_i' = \widehat{\theta_i} + \Delta\theta_i$. This is done multiple times with $\Delta\theta_i$ being progressively larger, until $J(\theta)|_{\theta_i=\theta_i'}$ satisfies . When this is the case, the current θ_i' corresponds to the upper boundary of the confidence interval of θ_i. The lower bound can be found by using negative values for $\Delta\theta_i$. A bisection method may be used to find the proper $\Delta\theta_i$, as is detailed in the Supplementary Materials of [15].

4. Identified Parameters from Single-Analyte Experiments and Three-Mixture Data Sets

Data set	Compound	Temperature (°C)												R_{max}
		12			16			20			24			
		k_a	k_d	K_A	k_a	k_d	K_A	k_a	k_d	K_A	k_a	k_d	K_A	
**Single-Analytes**	CBS	1.181<br>± 0.001	0.8954<br>± 0.0003	1.319<br>±0.001	1.434<br>± 0.002	1.4227<br>± 0.0007	1.008<br>±0.001	1.673<br>± 0.003	2.265<br>± 0.001	0.739<br>±0.001	2.011<br>± 0.004	3.438<br>± 0.003	0.585<br>±0.001	22.911<br>± 0.006
	BDS	4.715<br>± 0.008	4.166<br>± 0.003	1.132<br>±0.002	6.06<br>± 0.01	6.092<br>± 0.005	0.995<br>±0.002	7.84<br>± 0.02	8.836<br>± 0.009	0.887<br>±0.002	9.73<br>± 0.03	12.43<br>± 0.02	0.783<br>±0.003	24.25<br>± 0.01
	Sulfanilamide	0.714<br>± 0.001	3.456<br>± 0.003	0.2065<br>±0.0004	0.895<br>± 0.002	5.329<br>± 0.005	0.1680<br>±0.0004	1.118<br>± 0.003	8.114<br>± 0.009	0.1378<br>±0.0004	1.368<br>± 0.004	12.43<br>± 0.02	0.1101<br>±0.0004	25.81<br>± 0.01
	Furosemide	2.118<br>± 0.002	1.3745<br>± 0.0004	1.541<br>±0.001	2.641<br>± 0.003	1.9406<br>± 0.0007	1.361<br>±0.001	3.207<br>± 0.004	2.744<br>± 0.001	1.169<br>±0.001	3.877<br>± 0.005	3.776<br>± 0.002	1.027<br>±0.001	40.080<br>± 0.007
**B-C-D**	CBS	1.054<br>± 0.007	0.825<br>± 0.002	1.28<br>±0.01	1.14<br>± 0.01	1.217<br>± 0.004	0.933<br>±0.009	1.52<br>± 0.02	2.08<br>± 0.01	0.73<br>±0.01	1.64<br>± 0.03	2.95<br>± 0.02	0.56<br>±0.01	24.85<br>± 0.03
	BDS	5.199<br>± 0.009	3.99<br>± 0.01	1.304<br>±0.004	6.45<br>± 0.01	5.59<br>± 0.02	1.154<br>±0.004	8.29<br>± 0.02	8.65<br>± 0.03	0.958<br>±0.004	9.99<br>± 0.03	11.27<br>± 0.06	0.887<br>±0.005	24.00<br>± 0.02
	Sulfanilamide	0.811<br>± 0.006	2.86<br>± 0.02	0.284<br>±0.003	1.161*<br>± 0.009	4.97<br>± 0.05	0.234<br>±0.003	1.17*<br>± 0.01	7.1<br>± 0.1	0.166<br>±0.003	1.89<br>± 0.03	12.0<br>± 0.2	0.158<br>±0.003	23.56<br>± 0.08
	Furosemide	2.135<br>± 0.004	1.3727<br>± 0.0008	1.555<br>±0.003	2.620<br>± 0.005	1.938<br>± 0.001	1.352<br>±0.003	3.203<br>± 0.007	2.715<br>± 0.002	1.180<br>±0.003	3.753<br>± 0.009	3.698<br>± 0.004	1.015<br>±0.003	41.03<br>± 0.02
**A-C-D**	CBS	1.15<br>± 0.01	0.851<br>± 0.003	1.35<br>±0.01	1.43<br>± 0.01	1.357<br>± 0.004	1.06<br>±0.01	1.76<br>± 0.02	2.15<br>± 0.01	0.82<br>±0.01	2.07<br>± 0.04	3.29<br>± 0.02	0.63<br>±0.01	24.44<br>± 0.02
	BDS	5.201<br>± 0.009	4.18<br>± 0.01	1.244<br>±0.004	6.25<br>± 0.01	5.77<br>± 0.02	1.082<br>±0.004	8.27<br>± 0.02	8.91<br>± 0.03	0.927<br>±0.004	9.91<br>± 0.03	11.72<br>± 0.05	0.846<br>±0.004	24.22<br>± 0.02
	Sulfanilamide	1.04<br>± 0.01	2.08<br>± 0.03	0.50<br>±0.01	1.67*<br>± 0.01	3.86<br>± 0.07	0.432<br>±0.008	1.67*<br>± 0.02	6.0<br>± 0.2	0.277<br>±0.008	2.34<br>± 0.04	9.4<br>± 0.3	0.249<br>±0.008	22.46<br>± 0.07
	Furosemide	2.080<br>± 0.003	1.3757<br>± 0.0008	1.512<br>±0.002	2.478<br>± 0.004	1.910<br>± 0.001	1.297<br>±0.002	3.089<br>± 0.006	2.701<br>± 0.002	1.143<br>±0.002	3.611<br>± 0.008	3.657<br>± 0.003	0.988<br>±0.002	41.68<br>± 0.02
**A-B-D**	CBS	1.309<br>± 0.004	0.874<br>± 0.001	1.499<br>±0.005	1.698<br>± 0.006	1.455<br>± 0.002	1.167<br>±0.005	1.96<br>± 0.01	2.251<br>± 0.004	0.870<br>±0.005	2.46<br>± 0.01	3.549<br>± 0.009	0.692<br>±0.004	23.80<br>± 0.01
	BDS	4.79<br>± 0.02	4.18<br>± 0.02	1.144<br>±0.007	6.46<br>± 0.03	5.90<br>± 0.03	1.096<br>±0.007	8.51<br>± 0.04	8.74<br>± 0.05	0.974<br>±0.007	10.45<br>± 0.05	11.25<br>± 0.07	0.929<br>±0.007	22.52<br>± 0.04
	Sulfanilamide	1.053*<br>± 0.009	3.43<br>± 0.02	0.307<br>±0.003	1.05*<br>± 0.01	5.28<br>± 0.05	0.200<br>±0.003	1.05*<br>± 0.02	7.19<br>± 0.09	0.147<br>±0.003	1.24<br>± 0.03	12.5<br>± 0.3	0.099<br>±0.003	27.9<br>± 0.1
	Furosemide	2.148<br>± 0.004	1.3904<br>± 0.0009	1.545<br>±0.003	2.526<br>± 0.005	1.910<br>± 0.001	1.323<br>±0.003	3.153<br>± 0.007	2.696<br>± 0.002	1.169<br>±0.003	3.654<br>± 0.009	3.638<br>± 0.003	1.005<br>±0.003	41.61<br>± 0.03

Data set	Compound	Temperature (^oC)												R_{max}
		12			16			20			24			
		k_a	k_d	K_A	k_a	k_d	K_A	k_a	k_d	K_A	k_a	k_d	K_A	
**A-B-C**	CBS	1.418 ± 0.005	0.887 ± 0.001	1.598 ±0.006	1.885 ± 0.008	1.493 ± 0.002	1.263 ±0.005	2.27 ± 0.01	2.407 ± 0.005	0.944 ±0.005	2.92 ± 0.02	3.78 ± 0.01	0.771 ±0.005	23.76 ± 0.02
	BDS	5.16 ± 0.01	4.21 ± 0.01	1.225 ±0.005	6.30 ± 0.02	5.72 ± 0.02	1.101 ±0.005	8.44 ± 0.02	8.51 ± 0.03	0.991 ±0.005	10.29 ± 0.03	11.27 ± 0.04	0.914 ±0.005	24.18 ± 0.02
	Sulfanilamide	0.816 ± 0.005	3.15 ± 0.03	0.259 ±0.003	1.106* ± 0.009	5.80 ± 0.07	0.191 ±0.003	1.11* ± 0.01	8.4 ± 0.1	0.131 ±0.003	1.55 ± 0.02	14.4 ± 0.3	0.108 ±0.003	23.99 ± 0.08
	Furosemide	2.000 ± 0.009	1.433 ± 0.002	1.396 ±0.007	2.21 ± 0.01	1.885 ± 0.003	1.170 ±0.006	2.64 ± 0.01	2.555 ± 0.004	1.032 ±0.005	3.02 ± 0.01	3.413 ± 0.006	0.885 ±0.005	43.95 ± 0.08

Table S1: Kinetic parameters identified from single- and multiple-analyte injections of mixtures of known composition. Multi-analyte fits were obtained by fitting the multi-analyte model independently using any combination of three mixtures (A-B-C-D). 95% confidence intervals are given underneath the identified parameters. The estimated affinity is also reported. Association rates (k_a) are reported in $10^4 s^{-1}M^{-1}$, dissociation rates (k_d) are reported in $10^{-2}s^{-1}$, affinities (K_A) are reported in 10^6M^{-1} and maximal responses (R_{max}) are reported in RU. A star (*) next to a parameter value indicates that a constraint on the corresponding parameter was active in the fit.

5. Thermodynamic Parameters Identified with Three-Mixture Data Sets

Data set	Compound	$\Delta H^*_{k_a}$ (kcal/mol)	$\Delta S^*_{k_a}$ (cal/mol/K)	$\Delta H^*_{k_d}$ (kcal/mol)	$\Delta S^*_{k_d}$ (cal/mol/K)	$\Delta H^0_{K_A}$ (kcal/mol)	$\Delta S^0_{K_A}$ (cal/mol/K)
**Single-Analytes**	CBS	6.79	-16.00	18.33	-3.52	-11.54	-12.47
	BDS	9.66	-3.19	14.78	-12.90	-5.13	9.71
	Sulfanilamide	8.57	-10.75	17.34	-4.34	-8.77	-6.41
	Furosemide	7.88	-11.02	13.69	-18.95	-5.82	7.93
**B-C-D**	CBS	6.28	-18.05	17.75	-5.76	-11.47	-12.30
	BDS	8.73	-6.27	14.39	-14.40	-5.66	8.13
	Sulfanilamide	10.13	-5.03	19.01	1.19	-8.88	-6.23
	Furosemide	7.39	-12.69	13.36	-20.12	-5.96	7.42
**A-C-D**	CBS	7.72	-12.79	18.46	-3.19	-10.74	-9.60
	BDS	8.74	-6.23	14.27	-14.71	-5.53	8.48
	Sulfanilamide	9.67	-6.03	20.32	5.19	-10.65	-11.23
	Furosemide	7.31	-13.05	13.23	-20.58	-5.91	7.54
**A-B-D**	CBS	7.97	-11.63	18.96	-1.34	-10.99	-10.29
	BDS	10.45	-0.35	13.57	-17.14	-3.12	16.79
	Sulfanilamide	1.43	-35.10	17.07	-5.31	-15.64	-29.79
	Furosemide	7.07	-13.86	13.02	-21.30	-5.95	7.44
**A-B-C**	CBS	9.31	-6.77	19.74	1.41	-10.43	-8.17
	BDS	9.37	-4.05	13.52	-17.36	-4.15	13.30
	Sulfanilamide	7.53	-14.10	20.16	5.44	-12.63	-19.55
	Furosemide	5.37	-19.98	11.66	-26.02	-6.29	6.03

Table S2 : Identified thermodynamic parameters obtained from single-analyte experiments and by fitting the multi-analyte model to any combination of three mixtures (A-B-C-D) at four temperatures. The activation enthalpy and activation entropy were obtained by linearizing the Eyring equation using the identified association and dissociation rates at 12, 16, 20 and 24°C for each analyte. They are given for both the association ($\Delta H^*_{k_a}$ and $\Delta S^*_{k_a}$) and dissociation ($\Delta H^*_{k_d}$ and $\Delta S^*_{k_d}$) pseudo-reactions. The standard reaction enthalpy and entropy were obtained by linearizing the Van't Hoff equation using the identified affinities of each analyte.

6. Importance of the Initial Estimates for Parameter Identification

Data set	Compound	Temperature (°C)												R_{max}
		12			16			20			24			
		k_a	k_d	K_A	k_a	k_d	K_A	k_a	k_d	K_A	k_a	k_d	K_A	
**Starting Point, our algorithm**	CBS	0.77	0.81	0.95	0.98	1.24	0.79	1.16	1.89	0.61	1.40	2.83	0.49	27.87
	BDS	5.63	4.69	1.20	6.95	6.30	1.10	8.22	8.40	0.98	9.76	11.11	0.88	23.18
	Sulfanilamide	0.98	2.53	0.39	1.22	4.32	0.28	1.22	7.28	0.17	1.88	12.09	0.16	23.24
	Furosemide	2.26	1.38	1.64	2.75	1.95	1.41	3.22	2.72	1.18	3.97	3.78	1.05	40.48
**Final estimate, our algorithm**	CBS	1.05<br>(10.8%)	0.83<br>(8.3%)	1.28<br>(2.7%)	1.14<br>(20.8%)	1.22<br>(14.3%)	0.93<br>(7.6%)	1.52<br>(9.0%)	2.08<br>(8.6%)	0.73<br>(0.4%)	1.64<br>(18.2%)	2.95<br>(14.3%)	0.56<br>(4.5%)	24.85<br>(8.5%)
	BDS	5.20<br>(10.3%)	3.99<br>(4.4%)	1.30<br>(15.4%)	6.45<br>(6.4%)	5.59<br>(8.2%)	1.15<br>(16.0%)	8.29<br>(5.7%)	8.65<br>(2.2%)	0.96<br>(8.1%)	9.99<br>(2.7%)	11.27<br>(9.4%)	0.89<br>(13.3%)	24.00<br>(1.0%)
**(χ^2:0.0247)**	Sulfanilamide	0.81<br>(13.6%)	2.86<br>(17.5%)	0.28<br>(37.6%)	1.16<br>(29.6%)	4.97<br>(6.8%)	0.23<br>(39.1%)	1.17<br>(4.6%)	7.10<br>(13.0%)	0.17<br>(20.2%)	1.89<br>(38.2%)	12.00<br>(3.6%)	0.16<br>(43.3%)	23.56<br>(8.7%)
	Furosemide	2.14<br>(0.8%)	1.37<br>(0.2%)	1.56<br>(0.6%)	2.62<br>(0.8%)	1.94<br>(0.1%)	1.35<br>(0.7%)	3.203<br>(0.1%)	2.715<br>(0.9%)	1.18<br>(0.8%)	3.75<br>(3.2%)	3.70<br>(2.2%)	1.01<br>(1.1%)	41.03<br>(2.4%)
**Suboptimal starting point**	CBS	1	1	1	1	1	1	1	1	1	1	1	1	10
	BDS	1	1	1	1	1	1	1	1	1	1	1	1	10
	Sulfanilamide	1	1	1	1	1	1	1	1	1	1	1	1	10
	Furosemide	1	1	1	1	1	1	1	1	1	1	1	1	10
**Final estimate, suboptimal start**	CBS	1.52<br>(18.1%)	4.94<br>(448.5%)	0.31<br>(78.6%)	2.69<br>(77.2%)	8.85<br>(519.2%)	0.30<br>(71.4%)	4.36<br>(147.8%)	14.59<br>(554.3%)	0.30<br>(62.1%)	5.31<br>(153.1%)	18.91<br>(451.5%)	0.28<br>(54.1%)	7.82<br>(64.7%)
	BDS	4.84<br>(6.4%)	4.01<br>(2.2%)	1.21<br>(4.3%)	5.88<br>(11.4%)	5.71<br>(5.7%)	1.03<br>(6.0%)	7.03<br>(12.4%)	8.27<br>(3.1%)	0.85<br>(9.5%)	8.37<br>(21.0%)	11.51<br>(7.5%)	0.73<br>(14.6%)	29.11<br>(26.0%)
**(χ^2:0.0301)**	Sulfanilamide	0.48<br>(30.8%)	1.05<br>(69.1%)	0.46<br>(123.9%)	0.61<br>(29.8%)	1.74<br>(67.4%)	0.35<br>(115.0%)	0.71<br>(35.6%)	2.77<br>(66.8%)	0.26<br>(94.0%)	1.20<br>(17.3%)	4.69<br>(64.4%)	0.26<br>(132.4%)	20.31<br>(19.1%)
	Furosemide	2.44<br>(12.4%)	1.23<br>(9.2%)	1.98<br>(23.8%)	3.05<br>(14.0%)	1.77<br>(7.7%)	1.72<br>(23.5%)	3.84<br>(13.4%)	2.56<br>(6.1%)	1.50<br>(20.7%)	4.56<br>(9.9%)	3.54<br>(7.1%)	1.29<br>(18.3%)	37.31<br>(6.5%)

Table S3: Kinetic parameters identified from multiple-analyte injections of mixtures of known composition. Multi-analyte fits were obtained by fitting the multi-analyte model using mixtures B, C and D. Starting points and final estimates for part 2 of the suggested algorithm are reported whether part 1 was performed prior (our algorithm) or not (suboptimal starting point, expected order of magnitude of the parameters). The estimated affinity is also reported. Association rates (k_a) are reported in $10^4 s^{-1} M^{-1}$, dissociation rates (k_d) are reported in $10^{-2} s^{-1}$, affinities (K_A) are reported in $10^6 M^{-1}$ and maximal responses (R_{max}) are reported in RU. χ^2 values are reported below the data set name, in parentheses.

7. Identified Parameters from Two-Mixture Data Sets

Data set	Compound	Temperature (^{o}C)												R_{max}
		12			16			20			24			
		k_a	k_d	K_A	k_a	k_d	K_A	k_a	k_d	K_A	k_a	k_d	K_A	
**A-B**	CBS	1.55 ± 0.01	0.923 ± 0.002	1.69 ±0.01	2.04 ± 0.02	1.500 ± 0.003	1.36 ±0.01	2.55 ± 0.02	2.484 ± 0.008	1.03 ±0.01	3.07 ± 0.03	3.70 ± 0.01	0.831 ±0.009	24.2 ± 0.1
	BDS	4.18 ± 0.07	4.11 ± 0.02	1.02 ±0.02	6.04 ± 0.07	6.45 ± 0.04	0.94 ±0.01	8.21 ± 0.08	9.12 ± 0.06	0.90 ±0.01	10.2 ± 0.1	11.90 ± 0.09	0.86 ±0.01	19.6 ± 0.2
	Sulfanilamide	1.36 ± 0.03	3.65 ± 0.02	0.373 ±0.009	1.42 ± 0.03	4.65 ± 0.03	0.304 ±0.006	1.45 ± 0.04	6.60 ± 0.06	0.220 ±0.006	1.70 ± 0.05	9.4 ± 0.1	0.181 ±0.005	32.3 ± 0.4
	Furosemide	1.51 ± 0.04	1.370 ± 0.005	1.10 ±0.03	1.64 ± 0.04	1.854 ± 0.006	0.89 ±0.02	2.02 ± 0.05	2.402 ± 0.008	0.84 ±0.02	2.38 ± 0.06	3.33 ± 0.01	0.71 ±0.02	46.9 ± 0.7
**A-C**	CBS	1.390 ± 0.008	0.882 ± 0.002	1.575 ±0.009	1.71 ± 0.01	1.435 ± 0.004	1.194 ±0.01	1.88 ± 0.05	2.27 ± 0.02	0.83 ±0.02	2.86 ± 0.04	3.77 ± 0.02	0.76 ±0.01	23.91 ± 0.02
	BDS	5.18 ± 0.01	4.26 ± 0.02	1.215 ±0.005	6.28 ± 0.02	5.65 ± 0.02	1.113 ±0.005	8.61 ± 0.03	8.59 ± 0.04	1.003 ±0.005	10.31 ± 0.03	11.30 ± 0.05	0.912 ±0.005	24.25 ± 0.02
	Sulfanilamide	0.865 ± 0.009	2.91 ± 0.06	0.298 ±0.007	1.44 ± 0.01	4.8 ± 0.1	0.297 ±0.007	1.46 ± 0.05	5.0 ± 0.2	0.29 ±0.02	1.56 ± 0.05	12.8 ± 0.6	0.122 ±0.007	23.2 ± 0.1
	Furosemide	2.01 ± 0.01	1.433 ± 0.002	1.402 ±0.009	2.23 ± 0.01	1.887 ± 0.004	1.184 ±0.008	2.66 ± 0.02	2.562 ± 0.005	1.039 ±0.007	3.04 ± 0.02	3.413 ± 0.007	0.891 ±0.006	43.74 ± 0.09
**A-D**	CBS	1.304 ± 0.008	0.872 ± 0.002	1.50 ±0.01	1.62 ± 0.01	1.426 ± 0.004	1.138 ±0.009	1.86 ± 0.02	2.20 ± 0.01	0.847 ±0.01	2.42 ± 0.03	3.52 ± 0.02	0.688 ±0.008	23.98 ± 0.02
	BDS	5.04 ± 0.02	4.09 ± 0.03	1.231 ±0.009	6.58 ± 0.03	5.39 ± 0.04	1.221 ±0.009	8.58 ± 0.04	7.79 ± 0.06	1.102 ±0.009	10.42 ± 0.05	10.55 ± 0.09	0.99 ±0.01	23.42 ± 0.05
	Sulfanilamide	1.02 ± 0.01	2.84 ± 0.05	0.358 ±0.008	1.28* ± 0.02	4.9 ± 0.1	0.260 ±0.007	1.28* ± 0.03	6.6 ± 0.2	0.193 ±0.008	1.57 ± 0.04	14.1 ± 0.6	0.112 ±0.005	24.8 ± 0.1
	Furosemide	2.060 ± 0.006	1.381 ± 0.001	1.491 ±0.004	2.398 ± 0.007	1.886 ± 0.002	1.271 ±0.004	2.970 ± 0.009	2.660 ± 0.003	1.117 ±0.004	3.49 ± 0.01	3.618 ± 0.004	0.966 ±0.003	42.16 ± 0.04
**B-C**	CBS	1.31 ± 0.01	0.847 ± 0.003	1.55 ±0.01	1.31 ± 0.02	1.272 ± 0.006	1.03 ±0.01	1.34 ± 0.03	2.05 ± 0.02	0.65 ±0.02	1.77 ± 0.05	3.11 ± 0.03	0.57 ±0.02	23.29 ± 0.06
	BDS	5.02 ± 0.01	4.29 ± 0.02	1.171 ±0.006	6.61 ± 0.02	5.78 ± 0.03	1.144 ±0.006	8.75 ± 0.03	8.49 ± 0.04	1.031 ±0.006	10.59 ± 0.04	11.09 ± 0.07	0.955 ±0.007	23.61 ± 0.03
	Sulfanilamide	0.886 ± 0.008	3.14 ± 0.03	0.282 ±0.004	1.00* ± 0.01	5.23 ± 0.06	0.191 ±0.003	1.00* ± 0.01	6.18 ± 0.08	0.162 ±0.003	1.53 ± 0.02	12.2 ± 0.2	0.125 ±0.003	25.6 ± 0.1
	Furosemide	2.22 ± 0.02	1.465 ± 0.003	1.52 ±0.01	2.60 ± 0.02	1.953 ± 0.004	1.33 ±0.01	3.00 ± 0.02	2.623 ± 0.006	1.145 ±0.009	3.45 ± 0.03	3.543 ± 0.009	0.974 ±0.008	42.8 ± 0.1

Data set	Compound	Temperature (^oC)												R_{max}
		12			16			20			24			
		k_a	k_d	K_A	k_a	k_d	K_A	k_a	k_d	K_A	k_a	k_d	K_A	
**B-D**	CBS	1.08 ± 0.01	0.822 ± 0.003	1.31 ±0.02	1.13 ± 0.02	1.247 ± 0.007	0.91 ±0.02	1.75 ± 0.03	2.12 ± 0.01	0.83 ±0.02	1.89 ± 0.06	3.14 ± 0.03	0.60 ±0.02	24.61 ± 0.06
	BDS	5.28 ± 0.02	3.96 ± 0.02	1.331 ±0.009	7.12 ± 0.03	5.63 ± 0.03	1.27 ±0.01	7.68 ± 0.05	8.1 ± 0.1	0.95 ±0.01	10.38 ± 0.08	11.7 ± 0.1	0.89 ±0.01	23.89 ± 0.05
	Sulfanilamide	0.77* ± 0.01	2.89 ± 0.04	0.267 ±0.006	0.77* ± 0.02	4.4 ± 0.1	0.177* ±0.006	1.56 ± 0.03	8.8 ± 0.3	0.177* ±0.006	1.57 ± 0.04	10.6 ± 0.4	0.148 ±0.006	23.6 ± 0.2
	Furosemide	2.117 ± 0.007	1.371 ± 0.001	1.544 ±0.005	2.558 ± 0.009	1.911 ± 0.002	1.339 ±0.005	3.13 ± 0.01	2.720 ± 0.003	1.150 ±0.005	3.71 ± 0.02	3.696 ± 0.005	1.003 ±0.005	41.31 ± 0.05
**C-D**	CBS	0.99* ± 0.01	0.841 ± 0.004	1.18 ±0.01	0.99* ± 0.02	1.113 ± 0.007	0.89 ±0.01	0.99* ± 0.03	1.75 ± 0.02	0.57 ±0.02	0.99* ± 0.04	2.50 ± 0.03	0.40 ±0.02	23.2 ± 0.1
	BDS	4.97 ± 0.02	4.10 ± 0.01	1.210 ±0.007	6.75 ± 0.02	5.77 ± 0.02	1.171 ±0.006	8.90 ± 0.03	8.56 ± 0.04	1.041 ±0.006	11.19 ± 0.03	11.17 ± 0.06	1.002 ±0.006	22.34 ± 0.04
	Sulfanilamide	2.23* ± 0.09	2.87 ± 0.03	0.78* ±0.03	2.2* ± 0.1	2.87 ± 0.03	0.78* ±0.04	2.2* ± 0.1	3.54 ± 0.05	0.63 ±0.03	2.2* ± 0.1	3.72 ± 0.06	0.60 ±0.03	47 ± 1
	Furosemide	1.973 ± 0.009	1.304 ± 0.002	1.513 ±0.007	2.39 ± 0.01	1.889 ± 0.003	1.265 ±0.007	2.99 ± 0.02	2.686 ± 0.006	1.115 ±0.007	3.54 ± 0.02	3.762 ± 0.009	0.941 ±0.006	40.35 ± 0.06

Table S4: Kinetic parameters identified from multiple-analyte injections of mixtures of known composition. Multi-analyte fits were obtained by fitting the multi-analyte model independently using any combination of two mixtures (A-B-C-D). 95% confidence intervals are given underneath the identified parameters. The estimated affinity is also reported. Association rates (k_a) are reported in $10^4 s^{-1} M^{-1}$, dissociation rates (k_d) are reported in $10^{-2} s^{-1}$, affinities (K_A) are reported in $10^6 M^{-1}$ and maximal responses (R_{max}) are reported in RU. A star (*) next to a parameter value indicates that a constraint on the corresponding parameter was active in the fit.

8. Thermodynamic Parameters Identified with Two-Mixture Data Sets

Data set	Compound	$\Delta H^*_{k_a}$	$\Delta S^*_{k_a}$	$\Delta H^*_{k_d}$	$\Delta S^*_{k_d}$	$\Delta H^0_{K_A}$	$\Delta S^0_{K_A}$
**A-B**	CBS	8.97	-7.76	19.08	-0.83	-10.10	-6.92
	BDS	12.0	4.83	14.33	-14.45	-2.33	19.28
	Sulfanilamide	2.27	-31.62	12.78	-20.28	-10.51	-11.33
	Furosemide	6.04	-18.20	11.73	-25.83	-5.69	7.63
**A-C**	CBS	8.93	-8.25	19.69	1.20	-10.77	-9.45
	BDS	9.45	-3.76	13.50	-17.40	-4.05	13.64
	Sulfanilamide	7.02	-15.55	18.23	-1.64	-11.21	-13.92
	Furosemide	5.40	-19.86	11.67	-25.97	-6.27	6.11
**A-D**	CBS	7.79	-12.31	18.85	-1.76	-11.06	-10.56
	BDS	9.72	-2.81	12.93	-19.50	-3.20	16.70
	Sulfanilamide	4.96	-22.68	20.88	7.65	-15.92	-30.33
	Furosemide	6.99	-14.21	13.03	-21.28	-6.03	7.08
**B-C**	CBS	3.24	-28.37	17.85	-5.34	-14.62	-23.03
	BDS	10.05	-1.69	13.05	-18.95	-3.01	17.26
	Sulfanilamide	6.31	-18.35	17.26	-4.81	-10.94	-13.53
	Furosemide	5.60	-18.91	11.81	-25.44	-6.21	6.53
**B-D**	CBS	8.37	-10.75	18.60	-2.79	-10.23	-7.96
	BDS	8.28	-7.77	14.62	-13.62	-6.34	5.86
	Sulfanilamide	11.41	-0.79	18.87	0.70	-7.46	-1.49
	Furosemide	7.35	-12.88	13.43	-19.87	-6.08	6.99
**C-D**	CBS	-0.58	-42.19	15.04	-15.27	-15.62	-26.92
	BDS	10.86	1.14	13.73	-16.62	-2.88	17.76
	Sulfanilamide	-0.58	-40.58	3.59	-52.99	-4.16	12.41
	Furosemide	7.75	-11.62	14.28	-16.98	-6.53	5.36

Table S5: Identified thermodynamic parameters obtained from single-analyte experiments and by fitting the multi-analyte model to any combination of two mixtures (A-B-C-D) at four temperatures. The activation enthalpy and activation entropy were obtained by linearizing the Eyring equation using the identified association and dissociation rates at 12, 16, 20 and 24°C for each analyte. They are given for both the association ($\Delta H^*_{k_a}$ and $\Delta S^*_{k_a}$) and dissociation ($\Delta H^*_{k_d}$ and $\Delta S^*_{k_d}$) pseudo-reactions. The standard reaction enthalpy and entropy were obtained by linearizing the Van't Hoff equation using the identified affinities of each analyte.

9. Thermodynamic Parameters Identified with Two-Mixture Data Sets – Deviations from Single-Analyte Experiments

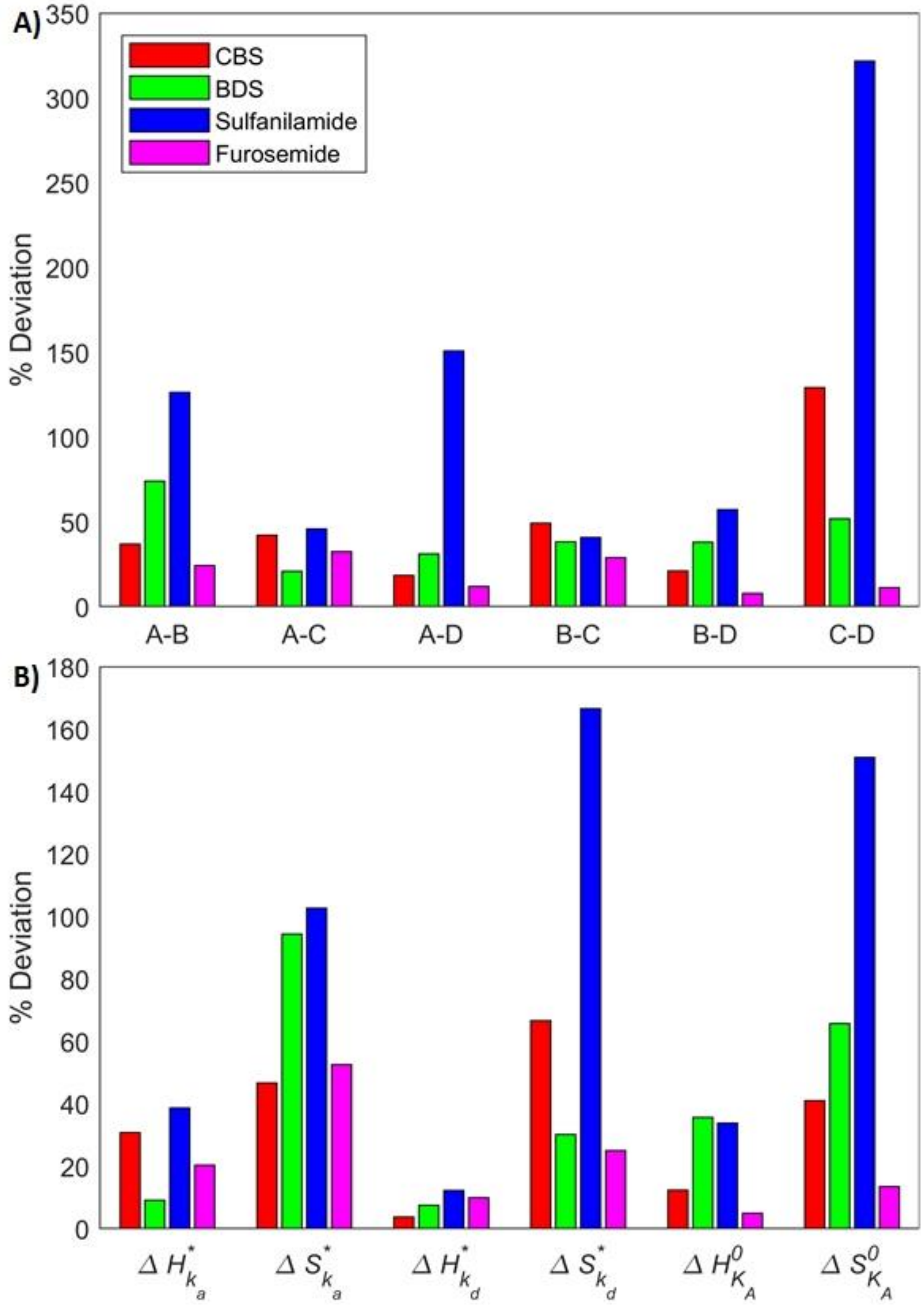

Figure S1: Deviation between the thermodynamic parameters ($\Delta H^*_{k_a}$, $\Delta S^*_{k_a}$, $\Delta H^*_{k_d}$, $\Delta S^*_{k_d}$, $\Delta H^0_{K_A}$ and $\Delta S^0_{K_A}$) derived from single-analyte experiments and parameters derived by fitting the multi-analyte model at four temperatures with any combination of two mixtures (A-B-C-D). A) Average deviation computed over all parameters for all analytes in all fits. B) Average deviation computed over all fits (except C-D) for all parameters of all analytes.

10. Composition Estimation when the Concentration is Known

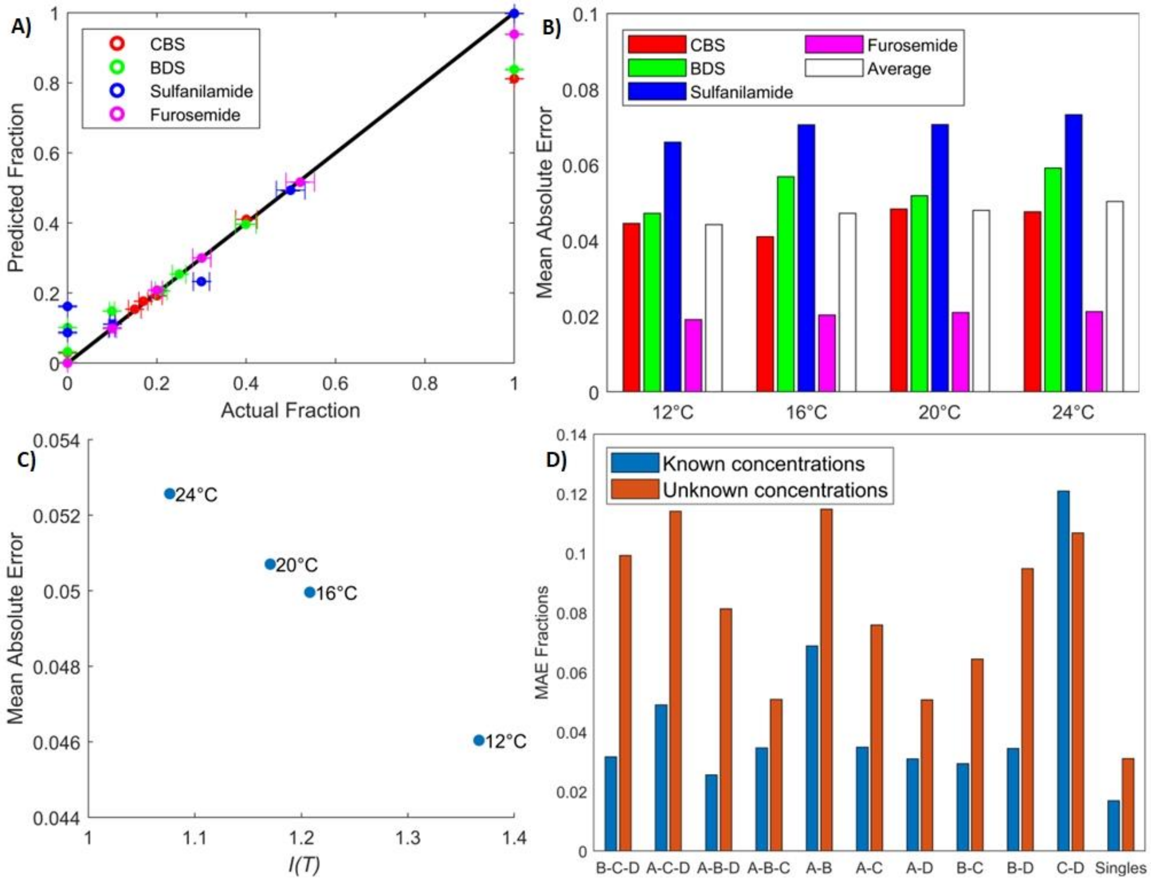

Figure S2: Performance of the composition estimation algorithm with known concentration. A) Calculated fractions with respect to actual fractions of the four analytes in each of the 8 mixtures detailed in . These fractions were estimated from sensorgrams at 12°C only with kinetic parameters identified from data set B-C-D. B) Mean absolute error of estimated fractions for each analyte at each temperature. The composition estimation algorithm was used independently for each set of kinetic parameters corresponding to each data set (all combinations of 2 and 3 mixtures) and each temperature, and mean absolute errors were averaged across all data sets. C) Mean absolute error (averaged across all data sets) with respect to the performance indicator. Annotations on the graph indicate the corresponding temperature. D) Mean absolute error of the fractions with respect to the data set that was used to identify the kinetics depending on if the concentration was considered known or added as a parameter to be estimated. Those were taken at 12°C only. In this figure, 'singles' refers to single-analyte fits.

11. Parameter Identification with Pooling

Data set	Compound	Temperature (°C)												R_{max}
		12			16			20			24			
		k_a	k_d	K_A	k_a	k_d	K_A	k_a	k_d	K_A	k_a	k_d	K_A	
**C-D**	CBS	1.07 ± 0.01	0.820 ± 0.003	1.30 ±0.01	1.07 ± 0.01	1.128 ± 0.006	0.95 ±0.01	1.53 ± 0.03	2.04 ± 0.02	0.75 ±0.01	1.55 ± 0.04	2.88 ± 0.03	0.54 ±0.02	22.01 ± 0.04
	BDS + Sulfanilamide	4.167 ± 0.006	3.80 ± 0.01	1.097 ±0.003	5.308 ± 0.009	5.33 ± 0.02	0.997 ±0.004	6.73 ± 0.02	8.34 ± 0.03	0.807 ±0.004	8.31 ± 0.02	10.98 ± 0.05	0.757 ±0.004	24.03 ± 0.02
	Furosemide	2.005 ± 0.004	1.345 ± 0.001	1.491 ±0.003	2.467 ± 0.006	1.912 ± 0.002	1.291 ±0.003	3.017 ± 0.007	2.679 ± 0.003	1.126 ±0.003	3.54 ± 0.01	3.649 ± 0.004	0.970 ±0.003	42.77 ± 0.04

Table S6: Kinetic parameters identified from multiple-analyte injections of mixtures of known composition. Multi-analyte fits were obtained by fitting the multi-analyte model using mixtures C and D. BDS and sulfanilamide were pooled into one analyte whose fraction consisted in the sum of the fractions of BDS and sulfanilamide. 95% confidence intervals are given underneath the identified parameters. The estimated affinity is also reported. Association rates (k_a) are reported in $10^4 s^{-1}M^{-1}$, dissociation rates (k_d) are reported in $10^{-2}s^{-1}$, affinities (K_A) are reported in 10^6M^{-1} and maximal responses (R_{max}) are reported in RU.

12. Structural Identifiability of Part 1 of the Parameter Identification Algorithm

The normalized dissociation phase signal of a given temperature is given by this sum of decaying exponentials:

$$R_{norm}(t) = \sum_{i=1}^{N} Z_i \exp\left(-k_{d,i} t\right)$$

Further detailing the definition of Z_i, we obtain:

$$R_{norm}(t) = \sum_{i=1}^{N} \frac{F_i K_{A,i} R_{max,i}}{K_{A,obs} R_{max,obs}} \exp\left(-k_{d,i} t\right)$$

With $K_{A,obs}$ and $R_{max,obs}$ calculated with the plateau values and thus known. The analyte fractions are also known and fed to the algorithm in the form of a M by N matrix in which every line corresponds to a mixture and every column corresponds to an analyte.

If only one mixture is available, the model is only locally identifiable, because multiple (but a finite number) of parameter sets lead to the same signal. This is because summation (here of exponential decays) is commutative. Indeed, the following two summations are equal:

$$R_{norm}(t) = \sum_{i=1}^{N} \frac{F_i K_{A,i} R_{max,i}}{K_{A,obs} R_{max,obs}} \exp\left(-k_{d,i} t\right) = \sum_{i=1}^{N} \frac{F_i K_{A,i}^* R_{max,i}^*}{K_{A,obs} R_{max,obs}} \exp\left(-k_{d,i}^* t\right)$$

With:

$$K_{A,i}^* R_{max,i}^* = K_{A,j} R_{max,j} \cdot \frac{F_j}{F_i}$$

$$k_{d,i}^* = k_{d,j}$$

If the js are not ordered the same way as the is, the exponentials are reordered without changing R_{norm}. This obfuscates the identification of $K_{A,i}$ and $R_{max,i}$ by a factor F_j/F_i in the following steps of the algorithm because the fractions F_i are known and their order remains unchanged. The N by 1 vector of identified dissociation rates would contain the correct elements, but in a different order. Hence the model is only locally identifiable.

To reach the same conclusion when two (or more) mixtures are available, the reordering needs to be the same in all mixtures. For example, if two mixtures are available, we have:

$$R_{norm,m_1}(t) = \sum_{i=1}^{N} \frac{F_{i,m_1} K_{A,i} R_{max,i}}{K_{A,obs,m_1} R_{max,obs,m_1}} \exp\left(-k_{d,i} t\right) = \sum_{i=1}^{N} \frac{F_{i,m_1} K_{A,i}^* R_{max,i}^*}{K_{A,obs,m_1} R_{max,obs,m_1}} \exp\left(-k_{d,i}^* t\right)$$

$$R_{norm,m_2}(t) = \sum_{i=1}^{N} \frac{F_{i,m_2} K_{A,i} R_{max,i}}{K_{A,obs,m_2} R_{max,obs,m_2}} \exp(-k_{d,i} t) = \sum_{i=1}^{N} \frac{F_{i,m_2} K_{A,i}^{*} R_{max,i}^{*}}{K_{A,obs,m_2} R_{max,obs,m_2}} \exp(-k_{d,i}^{*} t)$$

These equalities only hold if the following conditions are met, since $K_{A,i}$ and $R_{max,i}$ and $k_{d,i}$ are not mixture-dependent:

$$K_{A,i}^{*} R_{max,i}^{*} = K_{A,j} R_{max,j} \cdot \frac{F_{j,m_1}}{F_{i,m_1}} = K_{A,j} R_{max,j} \cdot \frac{F_{j,m_2}}{F_{i,m_2}}$$

$$k_{d,i}^{*} = k_{d,j}$$

Which may be simplified to:

$$\frac{F_{j,m_1}}{F_{i,m_1}} = \frac{F_{j,m_2}}{F_{i,m_2}}$$

$$k_{d,i}^{*} = k_{d,j}$$

Or:

$$\frac{F_{i,m_1}}{F_{i,m_2}} = \frac{F_{j,m_1}}{F_{j,m_2}}$$

$$k_{d,i}^{*} = k_{d,j}$$

In other words, if the fractions of any couple of analytes are perfectly correlated across all available mixtures, two or more parameter sets lead to the same signal and the model is only locally identifiable. This explains the added constraint on the required data described in section 4.2.1.2: $\frac{F_{i,m_1}}{F_{i,m_2}} \neq \frac{F_{j,m_1}}{F_{j,m_2}}$, or that the analyte fractions should not be perfectly correlated. This way, only one solution gives the signal that minimizes the objective function.

13. Identifiability of Part 1 of the Parameter Identification Algorithm

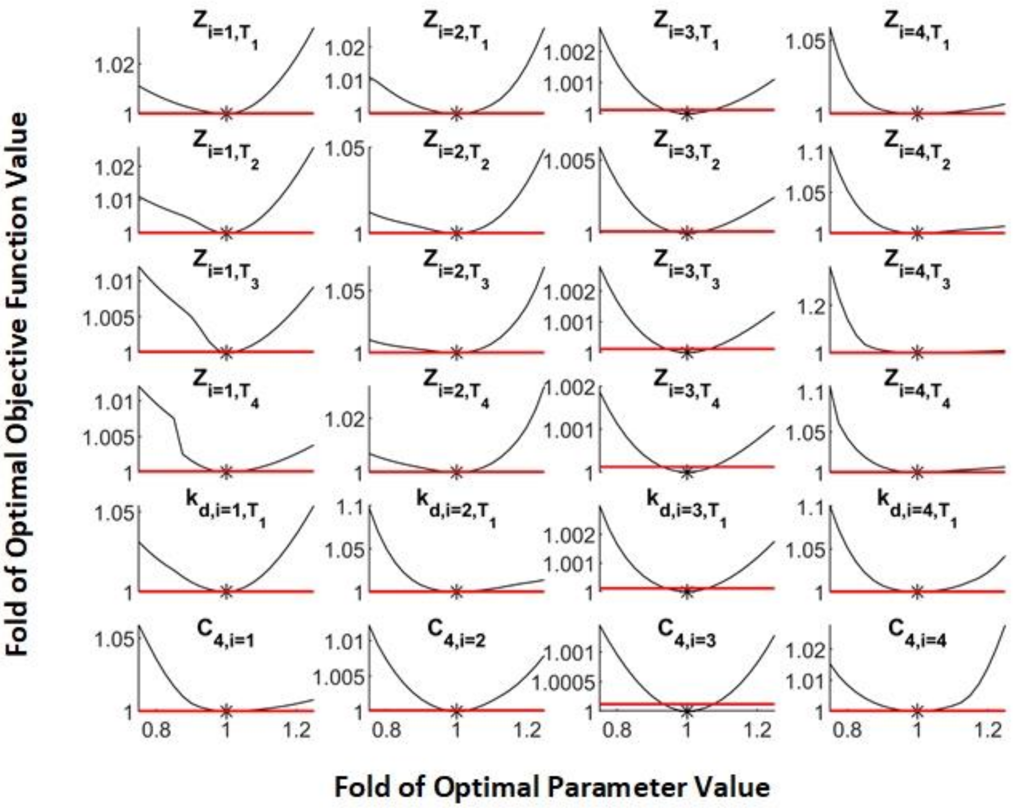

Figure S3: Profile likelihood of part 1 of the parameter identification algorithm with data set B-C. Each parameter was disturbed 21 times to fixed values ranging from 0.75 times to 1.25 times its optimal value (X-axis of the plots). For each disturbance, all the other parameters were reoptimized. The effect of disturbing each parameter on the value of the objective function is reported on the Y-axis of the plots. The red lines indicate the boundaries of the 95% confidence intervals of each parameter, as obtained with the method presented in section 0. A star (*) marks the location of the optimal fitted parameter. The confidence intervals of all parameters are finite in both directions.
